# The gut microbiome modulates the transformation of microglial subtypes

**DOI:** 10.1038/s41380-023-02017-y

**Published:** 2023-03-13

**Authors:** Yu Huang, Jing Wu, Hanping Zhang, Yifan Li, Lu Wen, Xunmin Tan, Ke Cheng, Yiyun Liu, Juncai Pu, Lanxiang Liu, Haiyang Wang, Wenxia Li, Seth W. Perry, Ma-Li Wong, Julio Licinio, Peng Zheng, Peng Xie

**Affiliations:** 1 NHC Key Laboratory of Diagnosis and Treatment on Brain Functional Diseases, The First Affiliated Hospital of Chongqing Medical University, Chongqing, 400016 China; 2 The Jin Feng Laboratory, Chongqing, 401329 China; 3 Department of Neurology, The First Affiliated Hospital of Chongqing Medical University, Chongqing, 400016 China; 4 Department of Neurology, Yongchuan Hospital of Chongqing Medical University, Chongqing, 402160 China; 5 Key Laboratory of Psychoseomadsy, Stomatological Hospital of Chongqing Medical University, Chongqing, 401147 China; 6 Department of Psychiatry, College of Medicine, SUNY Upstate Medical University, Syracuse, NY USA; 7 Institute for Brain Science and Disease, Chongqing Medical University, Chongqing, 400016 China

**Keywords:** Molecular biology, Cell biology

## Abstract

Clinical and animal studies have shown that gut microbiome disturbances can affect neural function and behaviors via the microbiota–gut–brain axis, and may be implicated in the pathogenesis of several brain diseases. However, exactly how the gut microbiome modulates nervous system activity remains obscure. Here, using a single-cell nucleus sequencing approach, we sought to characterize the cell type–specific transcriptomic changes in the prefrontal cortex and hippocampus derived from germ-free (GF), specific pathogen free, and colonized-GF mice. We found that the absence of gut microbiota resulted in cell-specific transcriptomic changes. Furthermore, microglia transcriptomes were preferentially influenced, which could be effectively reversed by microbial colonization. Significantly, the gut microbiome modulated the mutual transformation of microglial subpopulations in the two regions. Cross-species analysis showed that the transcriptome changes of these microglial subpopulations were mainly associated with Alzheimer’s disease (AD) and major depressive disorder (MDD), which were further supported by animal behavioral tests. Our findings demonstrate that gut microbiota mainly modulate the mutual transformation of microglial subtypes, which may lead to new insights into the pathogenesis of AD and MDD.

## Introduction

Substantial basic research has demonstrated that the gut microbiome can modulate neurochemistry and various behaviors through the microbiota–gut–brain (MGB) axis [1–3]. Clinical studies have found that major neuropsychiatric diseases such as Alzheimer’s disease (AD) [4], Parkinson’s disease [5], autism [6], and major depressive disorder (MDD) [7, 8] were accompanied by disturbances of the gut microbiome. Significantly, investigations further showed that certain microbial species were associated with the pathogenesis of representative neuropsychiatric diseases [9–13]. However, exactly how gut microbiota modulate molecular changes in the brain remains largely unknown. Uncovering this information is critical to understanding whether the gut microbiome might contribute directly to the pathology or treatment of brain diseases.

Germ-free (GF) mice, which are entirely devoid of gut microbiota, are an important model for studying the roles of the gut microbiome and the MGB axis [6, 10, 14–16]. Previously, several studies showed that gut microbiome absence altered hippocampus, prefrontal lobe, and amygdala transcriptomes, which were related to anxiety, depression, and cognitive impairment [17–20]. However, given the heterogeneity of neural cell types and their roles, bulk-tissue-level investigations may mask the complexity of changes across cells, and cannot interrogate the complex interplay between different cells [21]. In addition, previous studies highlighted that specific neural cell subpopulations were preferentially linked with the pathogenesis of neuropsychiatric diseases [22–25]. For example, Keren-Shaul et al. identified the disease-associated microglia (DAM) subtype, which was linked with AD [22]. Single-cell nucleus sequencing (snRNA-seq) approaches enable the characterization of cell type–specific transcriptome changes for tens of thousands of individual cells [26, 27]. The snRNA-seq studies have identified new potential pathophysiological mechanisms and intervention targets for neuropsychiatric diseases [28, 29], which highlight new avenues of investigation for how the gut microbiome affects the brain through the MGB axis.

To gain insight into cell type–specific transcriptomic changes, we performed unbiased snRNA-seq of the prefrontal lobe cortex (PFC) and hippocampus (Hip) derived from GF, specific pathogen free (SPF), and colonized-GF (CGF) mice. CGF mice were generated by co-housing juvenile (4-week-old) GF and SPF mice in flexible film gnotobiotic isolators for four weeks. We chose to investigate the PFC and Hip brain regions because: (i) the PFC and Hip are known principal targets of gut microbiome action on the brain via the MGB axis [15, 29, 30], and (ii) the PFC and Hip are closely linked with the development of neuropsychiatric disorders [31–33]. We found that the transcriptomes of microglia and their subpopulations were substantially modulated by the gut microbiome, which may have implications for the pathogenesis and treatment of AD and MDD.

## Method

### Ethics statement

The Ethical Committee of Chongqing Medical University (Chongqing, China; 2017013) reviewed the animal protocols and experiments. Kun Ming mice were used in all experiments. GF mice were obtained by sterile treatment on SPF mice in the NHC Key Laboratory of Diagnosis and Treatment on Brain Functional Diseases. The sterilization verification on GF mice was tested by sterile experiment on feces and skin, in accordance with Chinese Laboratory Animal-Microbiological Standards and Monitoring (GB 14922.2-2011). We followed the “Reduce, Reuse, Recycle” waste hierarchy, using the minimum sample size of 3 samples/group (*n* = 3/GF, *n* = 3/SPF, *n* = 3/CGF). This sample size is also commonly used in the single-cell analysis of murine brain tissues in previous studies [22, 28, 29, 34–37].

### Co-housing method

SPF mice were raised in different situations with GF and CGF. GF mice were labeled in 4 weeks after birth with an ear tag, then randomly allocated to GF or CGF by random number table. CGF mice were 4-week-old GF mice reared with SPF mice for 4 weeks in one GF isolator (4 CGF with 1 SPF/cage), see Supplementary Fig. 1a. GF and CGF mice were kept in flexible film gnotobiotic isolators until experiment and behavioral tests. All mice were housed on a 12-h light/dark cycle and had food and water ad libitum, with controlled temperature (22–24 °C) and (50–60%) humidity conditions. All mice were harvested at 8 weeks old.

### Extraction of single-cell nuclei

**Fig. 1:**
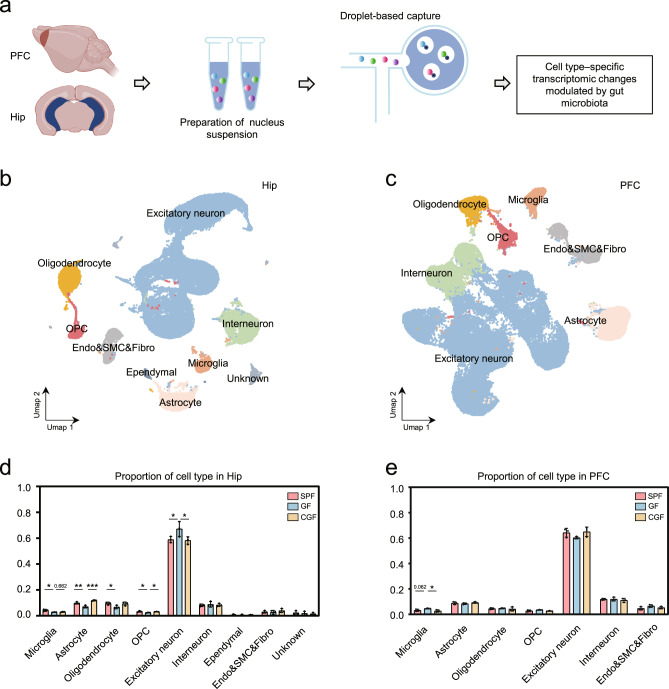
Gut microbiota absence changed microglial proportion in the Hip and PFC. **a** Schematic graph shows the workflow of acquiring hippocampus (Hip) and prefrontal lobe cortex (PFC) samples, nuclei isolation and droplet-based capture (10X Genomics) to produce cell type–specific transcriptomic signatures. **b**, **c** UMAP graph-based clustering and visualization of all captured nuclei for the Hip (**b**) and PFC (**c**). CNS resident cell types included excitatory neuron, interneuron, microglia, astrocyte, oligodendrocyte, OPC, ependymal cell, endothelial cell, smooth muscular cell, and fibroblast (Endo&SMC&Fibro). **d** In the Hip, GF mice had significantly decreased microglial proportion than SPF mice, and microbial colonization failed to rescue this change. The downregulated hippocampal astrocyte and OPC proportion in GF group relative to the SPF group were reversed by microbial colonization in the CGF group (SPF, *n* = 3; GF, *n* = 3; CGF, *n* = 3; data are mean ± SEM; GF vs. SPF, microglia, *P = 0.013, Excitatory neuron, *P = 0.0444, astrocyte, **P = 0.005, oligodendrocyte, *P = 0.0394, OPC, *P = 0.015; GF vs. CGF, microglia, *P* = 0.662, astrocyte, ***P = 0.0003, Excitatory neuron, *P = 0.0342, OPC, *P = 0.034; *P* values are from ANOVA post hoc analysis-LSD test). **e** In the PFC, there was an increased trend of proportion of microglia in GF group relative to SPF groups, which could be reversed by microbial colonization (SPF, *n* = 3; GF, *n* = 3; CGF, *n* = 3; data are mean ± SEM; GF vs. CGF, microglia, *P = 0.021; *P* values are from ANOVA post hoc analysis-LSD test).

The PFC and Hip were dissected, snap frozen in liquid nitrogen, then homogenized in 250 mM Sucrose, 10 mM Tris-HCl, 3 mM MgAc2, 0.1% Triton X-100 (Sigma-Aldrich, USA), 0.1 mM EDTA, 0.2U/μl RNase Inhibitor (Takara, Japan) (Fig. 1a), as previously described [38]. Nuclei were purified by sucrose gradient, and used at about 1000 nuclei/μl for snRNA-Seq.

### 10x library construction

The 10X Genomics Chromium Controller Instrument and Chromium Single Cell 3’V3.1 Reagent Kits (10X Genomics, Pleasanton, USA) were used to establish the 10x library. Nuclear suspensions (1000 nuclei/μl) were added to each channel to generate single-cell gel bead-in-emulsions (GEMs). This procedure breaks up GEMs, and purifies and amplifies barcoded-cDNA, after reverse transcription. The cDNA was then cut to make it fragmented, the A-end was loaded at the tail, the adapters joined, then index PCR amplification was performed. The final library was quantified using High Sensitivity DNA chip on a Bioanalyzer 2200 (Agilent, USA), and sequenced using a Novaseq 6000 (Illumina, USA) on a 150 bp paired-end run.

### Single-cell nucleus RNA statistical analysis

After applying the default filtering parameters (to exclude the adapted sequences and low-quality sequences) by using Fastp (v0.18.0), clean and high-quality sequence data were obtained. CellRanger v3.1.0 was used to get feature-barcode matrices through match reads to the mouse genome (mm10_Ensembl_Ensembl92). The aggregated matrix was obtained by downsample analysis of mapped barcoded reads per cell of each sample. Cells expressing fewer than 200 genes and cells with a mitochondrial UMI (Unique molecular identifiers) rate higher than 10% were excluded. Based on the above expression matrix, the Seurat package (version: 3.1.4) achieved the scaled data after cell normalization and regression [39, 40]. According to the scaled data, the top 2000 high-variable genes and top 10 principals were used to construct a principal component analysis (PCA) for uniform manifold approximation and projection for dimension reduction (UMAP). The unsupervised cell cluster was obtained based on the above PCA analysis and graph-based cluster method, and marker genes were calculated by FindAllMarkers (Wilcoxon) with default criteria, in line with single-cell approaches. Simultaneously, we used re-UMAP analysis, graph-based clustering, and marker analysis in the same cell type to find new cell subtypes.

### Differentially expressed genes burden analysis on downsampling data

Firstly, we identified differentially expressed genes (DEGs) among samples, and the function FindMarkers (Wilcoxon) was used under default criteria.

Next, we used a weighting algorithm that balanced the difference in the number of DEGs caused by unequal cell counts to explore preferentially influenced cell types in GF mice, as previously described [41]. We then calculated the number of DEGs by randomly sampling 1992 cells ten times (Student’s *t*-test, two-tailed).

### Gene ontology and canonical pathway analyses

Gene ontology analysis and canonical pathways analysis were conducted by GO (geneontology.org) and Ingenuity Pathway Analysis software (Qiagen, Shanghai, China), cutoff as |–logFDR| > 1.31 and |–log*P*| > 1.31.

### Pseudotime analysis

After selecting marker genes by the Seurat clustering result, Monocle2 was used to analyze Single-Cell Trajectories using DDR-tree with default parameters [42]. Finally, branch expression analysis modeling (BEAM analysis) was applied for branch fate-determined gene analysis.

### QuSAGE analysis (gene enrichment analysis)

A customized 41-signature gene set including immune-, cytokine-, and neurobiology-related terms was collected from the CellphoneDB database [43, 44], the neurotransmitters receptor gene of Genebank (https://www.ncbi.nlm.nih.gov/genbank/), and the immune scoring gene set as previously described [45]. QuSAGE analysis was used to quantify the relative activation of a given gene set, using QuSAGE (version 2.16.1). Full probability density function was used to show gene set differential expression levels, and significant enrichment pathway (*P* value < 0.05) analysis was used to visualize data.

### Cell communication analysis

The CellPhoneDB database [44] (cellphonedb V1.10 R package) analyzed cell-to-cell molecular communications in the Hip and PFC. Membrane, secreted, and peripheral proteins of the cluster of different time points were annotated. The mean counts of cell communication and its significance (*P* value < 0.05) were calculated based on the interactions.

### Behavioral tests

Prior to all behavioral tests, the experimental 8-week-old GF and CGF were removed from the sterile chamber and placed in a quiet test room for at least 1 h before test initiation, as previously described [7]. Tests were conducted during 9:00 am to 17:00 pm, under the same conditions as the typical breeding environment. All tests were video recorded and Noldus and ANY-maze software were used for data analyses. Behavioral videos were recorded and analyzed by an observer blinded to the experimental groups. Mice were tested in the open field test (OFT), forced swim test (FST) and Y-maze test.

OFT [46]: mice were individually placed in an uncovered cube of 45 cm length × 45 cm width and 45 cm height, with a black background. The mouse was placed in a corner of the box. After 5 min of adaptation, 5-min video was collected for analysis. The total distance traveled was used to evaluate the mouse’s locomotor activity.

FST [47]: cylinder tanks of 30 cm height and 15 cm diameter, containing 15 cm of water level at 24 °C were used. Mice were placed vertically, wetting all the hair, then the video was collected for 6 min. Duration of immobility during the middle 5 min of the total 6 min was used to evaluate the behavior of despair.

Y-maze test [48]: The Y-maze test instrument was composed of three open arms each 45 cm long, 10 cm wide and 29 cm height in a black background. Each mouse was placed in the same starting arm at the beginning of the experiment. The free exploration trajectory of the mouse was recorded for 8 min, and the order and times of entering the three open arms were used to calculate the spontaneous alternation rate (%) using the following equation = (the number of triads in turn) / (the total number of arm entries – 2) × 100%.

### Statistical methods and reproducibility

One-way ANOVA measured the proportion of cell types and numeric data of behavioral tests. Significance between the two groups was calculated by post hoc analysis with the least significant difference test (LSD) or Tamhane T2 test (when it was uneven variance). Detailed information on statistical procedure including variance and mean square is listed in Supplementary Table S5.

## Results

### Single-cell nucleus RNA-seq profiling of Hip and PFC

A schematic of nuclei isolation and the snRNA-seq workflow from the Hip and PFC is shown in Fig. 1a. Using the droplet-based single-nucleus method, we captured 72,226, and 67,698 nuclei from the Hip and PFC, respectively, in the 9 mice (3 per group). We obtained an average of 45,455 reads per nuclei in the Hip and 50,245 reads in PFC after stringent quality control (Supplementary Table S1).

After dimensionality reduction and graph-based clustering (UMAP), we identified 36 distinct clusters in the Hip (Supplementary Fig. 1b) and 29 clusters in PFC (Supplementary Fig. 1c). Then we annotated major cell types in the two regions based on the expression of well-established marker genes [49]. Here, excitatory neurons (*n* = 43,956 and 41,837 in the Hip and PFC, respectively; marked by *Grin2a*, *Syt1*, *Grin1*), interneurons (*n* = 6036, 7702; *Gad1*, *Gad2*), oligodendrocyte (*n* = 6021, 2984; *Plp1*, *Mog*, *Mbp*), OPC (Oligodendrocyte precursor cells; *n* = 2089, 1994; *Pdgfra*, *Vcan*), microglia (*n* = 2343, 2353; *Csf1r*, *Ctss*, *C1qa*), and astrocytes (*n* = 6803, 5862; *Aqp4*, *Gja1*) were clearly identified (Supplementary Fig. 1d, e and Fig. 1b, c).

### The absence of gut microbiota changed glial cells proportion in the Hip and PFC

Initially, we calculated the proportion of major cell types in two brain regions. In Hip, we found that the microglial proportion was significantly lower in GF compared to SPF (*P* = 0.013), and microbial colonization failed to rescue this change (GF vs. CGF, *P* = 0.662; Fig. 1d). Meanwhile, hippocampal astrocyte, oligodendrocyte and OPC proportion were downregulated in the GF group relative to the SPF group (GF vs. SPF, *P* = 0.005 for astrocyte, 0.0394 for oligodendrocyte and 0.015 for OPC), astrocyte and OPC were reversed by microbial colonization in the CGF group (GF vs. CGF, *P* = 0.0003, 0.034), excluded oligodendrocyte. Furthermore, in contrast to glial cells, excitatory neuron increased in GF (GF vs. SPF, *P* = 0.0444), microbial colonization reversed this trend in CGF (GF vs. CGF, *P* = 0.0342). In PFC, the proportion of microglia in the GF group relative to the SPF group trended upward (*P* = 0.062), which could be reversed by microbial colonization (Fig. 1e). We did not find any difference in the composition ratio of the remaining cell types between the three groups. These observations demonstrate that the presence or absence of the gut microbiome primarily impacted the relative composition of glia in the Hip and PFC.

### The absence of gut microbiota resulted in cell-specific transcriptomic changes

Next, we performed a DEGs analysis between GF and SPF (Supplementary Fig. 2a, b). We identified 4999 and 6122 DEGs across the major six cell types in the Hip and PFC, respectively, based on the total captured gene in each type (Supplementary Table S6 and Supplementary Fig. 2c). In these two brain regions, glial cells had more DEGs than neurons (Supplementary Fig. 2d, e). The top DEGs in the two regions were mainly involved in mitochondrial dysfunction and the RNA translation process even across cell types (Supplementary Fig. 2f, g).

**Fig. 2:**
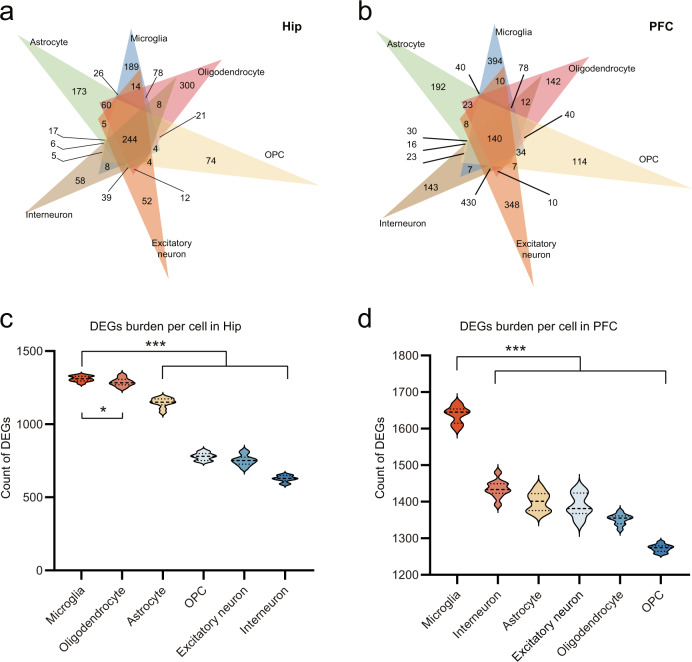
Gut microbiota mainly affected microglia in the two brain regions. **a**, **b** Venn diagrams describe 846 and 1333 cell-specific DEGs across six major cell types in the Hip (**a**) and PFC (**b**). **c** DEGs (GF vs. SPF) downsampling analysis showed greater microglial gene dysregulations in the Hip (10 times repetition; Microglia vs Astrocyte, ***P = 4.9471E–11, Microglia vs Oligodendrocyte, *P = 0.0395, Microglia vs OPC, ***P = 3.4168E–21, Microglia vs Excitatory neuron, ***P = 1.0866E–19, Microglia vs Interneuron, ***P = 6.7238E–24; *P* values are from two-tailed Student’s *t*-test). **d** DEGs (GF vs. SPF) downsampling analysis showed greater microglial gene dysregulations in PFC, Microglia vs Astrocyte, ***P = 2.5005E–14, Microglia vs Oligodendrocyte, ***P = 1.1953E–17, Microglia vs OPC, *P = 4.019E–20, Microglia vs Excitatory neuron, ***P = 1.1021E–13, Microglia vs Interneuron, ***P = 1.562E–13; 10 times repetition; *P* values are from two-tailed Student’s *t*-test).

To further uncover the cell-specific transcriptomic changes modulated by gut microbiota, we identified 846 and 1333 cell-specific DEGs across the six major cell types in the Hip and PFC (Fig. 2a, b), respectively. This observation highlights that the single-cell-level resolution is vital to uncover how the gut microbiome modulates transcriptional changes in the brain. The function enrichment pathways of these cell-specific genes were also significantly different. For example, the altered microglial cell-specific DEGs were enriched for neuroinflammatory and complement system signaling pathways in the Hip (Supplementary Fig. 3a), such as alterations of chemokine receptor-*Cx3cr1*, interferon gamma receptor*-Ifngr1*, interleukin receptor-*Il6ra*, and complement family-*C1qa*, *C1qb*, and *C1qc*, respectively. In contrast, in the PFC, microglial cell-specific DEGs were enriched for RhoGDI and IL-8 signaling pathways (Supplementary Fig. 3b), for example, *Map2k1, Nfatc3*, and *Rhot1*. Together, our DEGs analysis showed that the absence of gut microbiota resulted in cell-specific transcriptomic changes.

### Microglial transcriptomes were preferentially influenced

Here we explored which cell types were preferentially modulated by the gut microbiome. Disregarding different cellular counts, oligodendrocytes, astrocytes, and microglia mainly contributed to DEGs detected in the Hip (Supplementary Fig. 2d), while the majority of DEGs in PFC were derived from excitatory neurons, interneurons, and microglia (Supplementary Fig. 2e). To rule out the inherent confounding effects of unequal captured cell ratios (neuron: glia ratio = 2.89:1 in PFC and 3.2:1 in Hip), the DEGs burden analysis [41] was carried out by comparing the same number of nuclei across all cell types for ten times by downsampling the data. Accordingly, we found that microglia had the largest number of DEGs in both the Hip and PFC (Fig. 2c, d), suggesting that microglia were preferentially impacted among the six major cell types in the two regions. These findings aligned with the disparate microglial ratios also found in the two brain regions.

We determined whether microglial DEGs were brain-specific. Venn diagram analysis showed that there were 370 genes shared in two regions, while 563 genes only changed in the Hip, and 694 in PFC (Supplementary Fig. 4a). Functionally, we found that the GF mice were enriched for mitochondrial dysfunction, oxidative phosphorylation, inflammasome pathway, and NRF2-mediated oxidative stress response, and depleted for synaptogenesis, synaptic long-term potentiation, and synaptic long-term depression signaling in the Hip. In the PFC, some pathways such as NRF2-mediated oxidative stress response were consistently enriched in GF relative to SPF mice. However, synaptic-related pathways such as the synaptogenesis signaling and long-term synaptic potentiation showed opposite changes in the PFC relative to Hip (Supplementary Fig. 4b). Our results suggest that microglial transcriptional changes caused by the gut microbiome vary in a brain region-specific manner.

### The gut microbiome mainly modulated microglia-astrocyte communication

We conducted CellPhoneDB database [44] (cellphonedb V1.10 R package) analysis to uncover potential ligand-receptor pairs between cells to understand how gut microbiome absence influenced communication between microglia and other major cell types. Detailed data are shown in Supplementary Table S2. Microglia communicated mostly with astrocytes, followed by oligodendrocytes, in the Hip of the SPF group. Similar cell-to-cell communications were also found in the GF group, but the lack of a gut microbiome in that group resulted in decreased interaction intensities. Microbial colonization slightly increased the microglia-astrocyte communication (Supplementary Fig. 5a). For example, we found diminished communication between microglial *Entpd1* to astrocytic *Adora1* in the GF group, which was restored in the CGF group. The CD39 (*Entpd1*) and *Adora1* pair can regulate neuronal activity via its participation in adenosine metabolism [50]. In the SPF group, cellular communication between microglia and other cells was weaker in the PFC than in Hip. Interestingly, lack of a gut microbiome led to significantly increased microglia-astrocyte communication, ranking first in the communication between microglia and other cells. Furthermore, microbial colonization failed to modulate the communication between microglia and other cells (Supplementary Fig. 5a). In summary, the gut microbiome mainly influenced microglia-astrocyte communication in the Hip and PFC of GF and CGF mice.

### Microbial colonization rescued microglial gene alterations

Here, we identified 933 and 1064 microglial DEGs by comparing the GF and SPF groups. Interestingly, we found that most of DEGs from these two groups (74.91% and 78.76%) could be rescued by microbial colonization. These rescued genes were associated with chemical synaptic transmission, and cell-cell adhesion in the Hip (Supplementary Table S3 and Supplementary Fig. 5b). For example, we observed six rescued cadherin family genes (e.g., *Cdh8*, *Cdh9*, *Cdh11* and *Cdh12*), mainly involving in cell adhesion, and seven rescued gamma-aminobutyric acid (GABA) receptor genes (e.g., *Gabarap, Gabarapl2, Gabra2, Gabrb1*). In addition, genes enriched in fundamental molecular processes like protein binding and transport, were rescued in the PFC by microbial colonization, such as 6 reversed genes (e.g., *Eif1, Eif1b, Eif2s3y, Eif4e, Eif4h*, and *Eif5a*) belonging to eukaryotic initiation factor (Supplementary Fig. 5c). Our findings suggest that microbial colonization effectively reversed microglial transcriptomic changes in the Hip and PFC.

### Gut microbiota modulated mutual transformation of microglial subpopulations

**Fig. 3:**
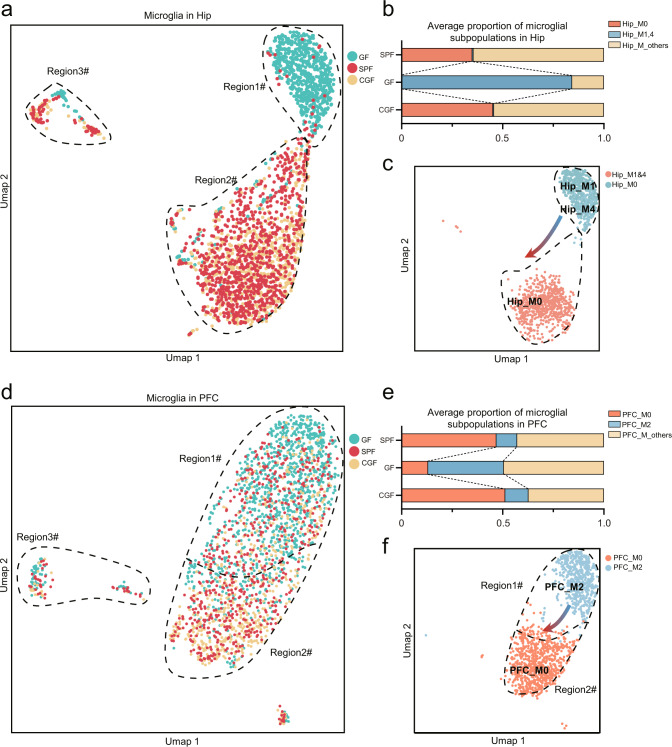
The gut microbiome modulates the mutual transformation between microglial subpopulations. **a** UMAP plot depicting microglial subpopulations divided by groups in the Hip. Graph clustering shows that GF cells were located primarily in region #1, and SPF and CGF cells were located in region #2. **b** Bar chart showing that subcluster 0 (Hip_M0) was enriched in SPF and CGF groups and diminished in GF group, subclusters 1 (Hip_M1) and 4 (Hip_M4) were enriched in the GF group and reversed by microbiome colonization. **c** UMAP plot depicting Hip_M1 and Hip_M4 located mainly at region #1, Hip_M0 at region #2. Combined with Pseudotime analysis (Supplementary Fig. 8a, c, e), the arrow showed a shifting trend of transforming relationships between Hip_M0, Hip_M1 and Hip_M4. **d** UMAP plot depicts microglial subpopulations divided by groups in the PFC. Graph clustering shows that GF cells contributed more plots in region #1 than SPF and CGF cells. Furthermore, cells in region #2 primarily belonged to SPF and CGF groups. **e** The bar chart showed that subcluster 0 (PFC_M0) was enriched in SPF and CGF groups and decreased in the GF group; subcluster 2 (PFC_M2) was enriched in the GF group and reversed by colonization. **f** UMAP plot depicted PFC_M2 mostly located at region #1, PFC_M0 at region #2. Combined with Pseudotime analysis (Supplementary Fig. 8 b, d, f), the arrow showed a shifting trend of transforming the relationship between PFC_M0 and PFC_M2.

Having demonstrated that gut microbiome absence mainly affects microglia, we wanted to further clarify if or how microglial subpopulations changed. Therefore, we performed a microglial re-clustering analysis, which yielded 10 and 6 subpopulations in the Hip and PFC, respectively. In the Hip, two microglial subpopulations (Hip_M1, M4), with a composition ratio of 83.98%, were significantly enriched in GF compared to SPF (0.53%), and microbial colonization could effectively reverse these changes in the CGF group (0.44%; *P* = 5.2523E–8, one-way ANOVA; Fig. 3a, b). For Hip_M0, a contrasting pattern was observed between the three groups (0.27% in GF, 34.99% in SPF, 45.18% in CGF; *P* = 0.014, one-way ANOVA; Fig. 3a, b). QuSAGE was used to identify functional gene sets of each subpopulation. This analysis showed that the anti-inflammatory and regulatory T cells (Treg) gene sets were most activated in Hip_M1 and Hip_M4 (Supplementary Fig. 6a–c), such as enrichment of *Entpd1, Mif* and *Tgfb1* (Supplementary Fig. 7a–d), which were inhibited in Hip_M0 (Supplementary Fig. 6, d).

In the PFC, the composition ratio of PFC_M2 (37.36%) was enriched in GF relative to SPF (10.23%), and could be effectively rescued in the CGF group (11.53%; *P* = 0.011 between the three groups, one-way ANOVA; Fig. 3d, e). Meanwhile, the composition ratio of PFC_M0 was significantly reduced in GF (13.10%) compared to SPF (46.85%) and CGF (51.19%), and enriched in SPF and CGF (46.85% in SPF, 51.19% in CGF; *P* = 0.015, one-way ANOVA; Fig. 3d, e). QuSAGE analysis showed that the anti-inflammatory and Treg gene sets, such as *Entpd1, Mif, Vegfa* and *Tgfb1* (Supplementary Fig. 7e, f), were most activated in PFC_M2 (Supplementary Fig. 6e, g), and inhibited in PFC_M0 (Supplementary Fig. 6e, f). Next, Pseudotime analysis was conducted to further explore these mutual transformational relationships. Hip_M1 and Hip_M4 were more located at the start of developmental trajectories; however, Hip_M0 was more located on the middle and end (Supplementary Fig. 8a, b). In PFC, PFC_M0 and PFC_M2 showed the same trend (Supplementary Fig. 8c, d). It suggested that gut microbiota modulate transformational control between different microglial subpopulations, displaying the shift from Hip_M1&4 to Hip_M0, and PFC_M2 to PFC_M0 (Fig. 3c, f). Together, these findings demonstrated that the gut microbiome modulated the mutual transformation of microglial subpopulations in the two regions.

To ensure these findings, we further carried out an independent snRNA-seq analysis of Hip among GF, SPF and CGF (*n* = 3/group), named batch 2 (B2). Generally, two batches of single-cell transcriptome data were highly consistent in the number of capturing cells (Supplementary Fig. 9a) and identification of the cell types (Supplementary Fig. 9b). In the analysis of microglia subtypes, we found that the three hippocampal microglial subpopulations observed in batch 2 were also highly similar to B1_Hip_M0, M1 and M4 (Supplementary Fig. 9c*;* Fisher’s exact test; Supplementary Table S7). Odd ratio of B2_Hip_M2 and M3 versus Hip_M0 were 133.6 (FDR = 3.00E–04) and 91.4 (FDR = 2.34E–05), B2_Hip_M0 versus Hip_M1 and M4 were 99.2 (FDR = 3.14E–89) and 27.7 (FDR = 3.90E–29). Furthermore, proportion of these subpopulations showed mutual transformation among groups (Supplementary Fig. 9d, e), B2_Hip_M2 and M3 increased in SPF and CGF, decreased in GF like B1_Hip_M0, moreover, B2_Hip_M0 showed opposite trend like B1_Hip_M1 and M4.

### The microglial genes rescued by microbial colonization are linked with AD and MDD

**Fig. 4:**
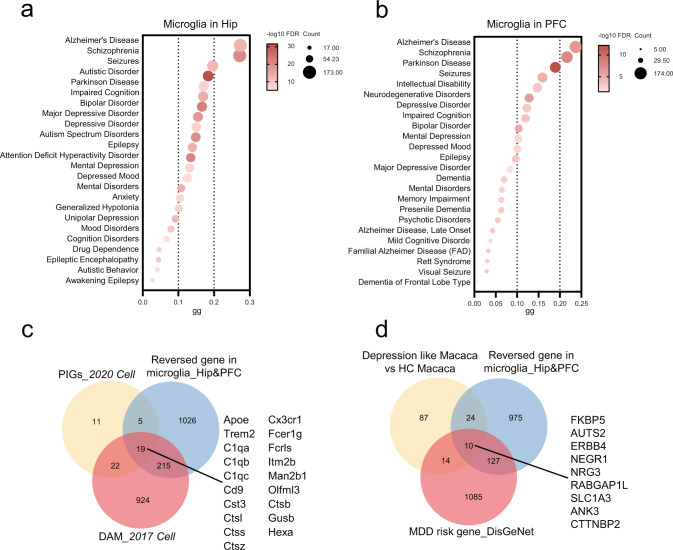
Microglial genes regulated by the gut microbiota are linked with AD and MDD. **a**, **b** In Hip (**a**) and PFC (**b**), reversed microglial genes were closed to neuropsychiatric diseases, such as AD and MDD. **c** 19 genes overlapped between plague-induced genes (PIGs), disease-associated microglia (DAM), and reversed genes, including *Apoe, Cx3cr1, Trem2, Fcer1g, C1qa, Fcrls, C1qb, Itm2b, C1qc, Man2b1, Cd9, Olfml3, Cst3, Ctsb, Ctsl, Gusb, Ctss, Hexa*, and *Ctsz*. **d** Ten genes overlapped between microglial DEGs in depression like macaque (unpublished data), MDD risk gene in the DisGeNet database and reversed genes in microglia (converted to HUGO Gene Symbol), including *FKBP5, AUTS2, ERBB4, NEGR1, NRG3, RABGAP1L, SLC1A3, ANK3, CTTNBP2*, and *ITGB5*.

To explore potential associations between the rescued genes and representative neuropsychiatric disorders, the DisGeNET database [51] was used for Disease Enrichment analysis. In both the Hip and PFC, the microglial genes rescued by microbial colonization were linked to neuropsychiatric diseases such as AD (*n* = 173 in Hip and 174 in PFC), MDD (*n* = 98 and 61), and autism (*n* = 116 and 52; Fig. 4a, b).

Single-cell studies of AD and MDD were selected to further confirm these findings. AD had the highest numbers of rescued microglial genes in the Hip and PFC, and we had a long-term interest in MDD. We found that, although the cell types associated with distinctive diseases were different, a large number of disease risk genes aligned with the microglial genes rescued by microbial colonization (Fig. 4a). In particular, for AD, 19 microglial genes overlapped between PIGs [52] (plague-induced genes), DAM [22], and reversed genes, including *Apoe, Fcer1g, C1qa, Frcls, C1qb, Itm2b, C1qc, Man2b1, Cd9, Olfml3, Cst3, Trem2, Ctsl, Ctsb, Ctss, Gusb, Ctsz, Hexa*, and *Cx3cr1* (Fig. 4d). As for MDD, we used our single-cell analysis of dorsolateral PFC from a non-human primate depression model (unpublished data, not shown), and matched them against MDD risk genes in the DisGeNet database. We found 10 overlapped genes, including *FKBP5, AUTS2, ERBB4, NEGR1, NRG3, RABGAP1L, SLC1A3, ANK3, CTTNBP2*, and *ITGB5* (Fig. 4e). These findings demonstrated that microglial genes reversed by microbial colonization were mainly linked with AD and MDD, suggesting that microbial modulation of these key microglial genes via the gut-brain axis may be a potential therapeutic strategy for AD and MDD.

### Cross-species analysis showed that microglia subpopulations regulated by gut microbiota were associated with AD and MDD

**Fig. 5:**
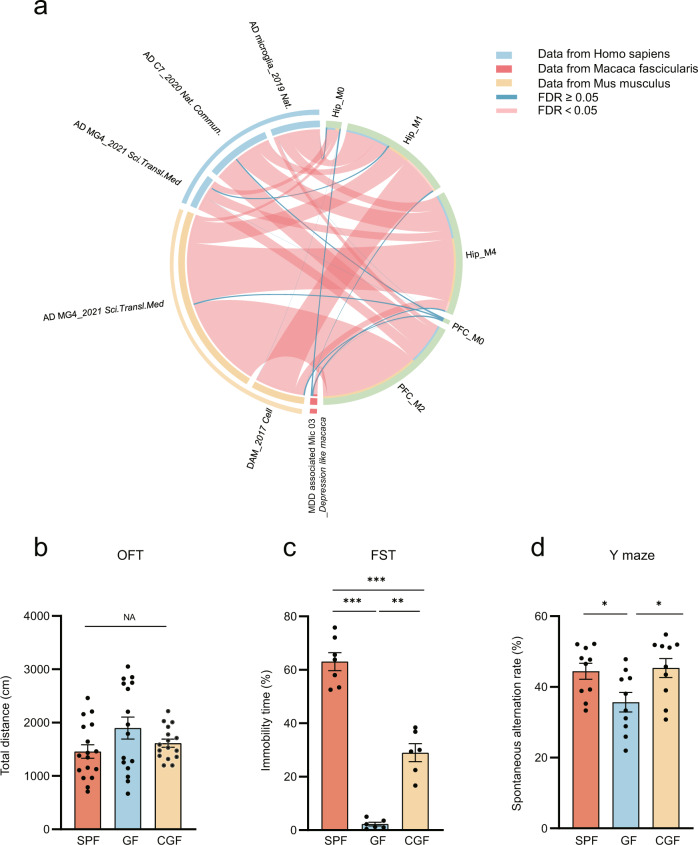
Cross-species analysis described that microglia subpopulations regulated by the gut microbiota were associated with AD and MDD. **a** Circos graph shows microglial subpopulations highly associated with AD and MDD cross humans, mice and macaca (proportion of segment means –logFDR, Fisher’s exact tests, FDR-BH corrected). **b** The OFT showed no differences in locomotion activity in the three groups (SPF, *n* = 17; GF, *n* = 16; CGF, *n* = 16; data are mean ± SEM; NA means *P* = 0.109; *P* values are from ANOVA test). **c** GF mice displayed decreased immobility time in FST and restored partially in CGF mice (*n* = 6 in GF and CGF, *n* = 7 in SPF; data are mean ± SEM; GF vs. SPF, ***P = 3.00E–06; GF vs. CGF, **P = 0.0011, SPF vs. CGF, ***P = 6.10E–05; *P* values are from ANOVA post hoc analysis-Tamhane T2 test). **d** The decrease of spontaneous alternation rate of GF mice in the Y-maze test compared to SPF mice was restored in CGF mice (*n* = 10/group; data are mean ± SEM; GF vs. SPF, *P = 0.022; GF vs. CGF, *P = 0.012; *P* values are from ANOVA post hoc analysis-LSD test).

Next, we further verified whether the transcriptomic changes of these 5 microglial subpopulations were linked with AD and MDD. We performed cross-species analysis of the association between these 5 microglial subpopulations and these two disorders by using animal and human sc/snRNA-seq data from five publications [22–24, 53], and snRNA-seq analysis from our non-human primate depression model (unpublished data, not shown). The marker genes of 5 microglia subpopulations were compared with the microglial marker genes or enriched DEGs associated with these diseases. We found that transcriptomic changes of these microglial subpopulations were highly associated with AD and MDD across human, mouse, and macaque (Fig. 5a). Furthermore, the noted DAM was highly similar to Hip_M1 (Fisher’s exact test, FDR = 3.52E–30, odd ratio = 7.379; Supplementary Table S4) and Hip_M4 (Fisher’s exact test, FDR = 3.32E–08, odd ratio = 3.896). In addition, we found that only PFC_M2 was significant relative to MDD-associated microglia in Macaca (Fisher’s exact test, FDR = 0.001, odd ratio = 2.605). This cross-species analysis provided evidence that microglia subpopulations’ transcriptomic changes modulated by gut microbiota were highly linked with AD and MDD.

### Behavioral tests support the association between gut microbiota, AD, and MDD

We used an animal behavioral test panel related to AD and MDD including OFT, Y-maze and FST to confirm the above findings. There was no difference in locomotion activity between the three groups (*P* = 0.109; Fig. 5b). However, the percent immobility time was significantly decreased in the FST of GF compared to SPF mice (*P* = 6.9879E–11), suggesting impacts on behavior despair. Microbial colonization increased the percent immobility time in CGF mice, although it was not completely restored to the same level as in SPF mice (Fig. 5c). In the Y-maze test, the spontaneous alternation rate was significantly decreased in GF relative to SPF (*P* = 0.023), and this change could be completely reversed by microbial colonization (CGF) (*P* = 0.013; Fig. 5d). This behavioral test suggested a close association between the gut microbiome and short-term memory changes. Together, our behavioral studies support the single-cell observations that transcriptomic changes of microglial subpopulations were highly linked with AD and MDD.

## Discussion

Our study showed that the absence of a gut microbiome significantly affected transcriptional changes at the single-cell level, especially in microglia and their subtypes. Microbial colonization could effectively reverse these transcriptional changes. Significantly, we found that the gut microbiome modulated mutual transformation of microglial subtypes. Cross-species analysis showed that transcriptomic changes of microglial subpopulations were linked with AD and MDD, and with animal behavior changes relevant to these diseases. Our findings provide new evidence for how the gut microbiome may regulate brain function through the MGB at single-cell molecular resolution. Furthermore, these findings may pave the way for a better understanding of the pathophysiological mechanisms of AD and MDD.

In recent years, important progress has been made in understanding how the gut microbiome affects the brain [1, 2], but its transcriptional changes at the single-cell level have not been uncovered. Here, we found that the lack of a gut microbiome can lead to systematic changes in the transcription of neuronal and glial cells. Among them, microglia were most vulnerable to the influence of the gut microbiome. In line with our findings, Erny et al. observed significant differences in microglial structure and function between SPF and GF mice [54], and Thion et al. found that signals from the maternal gut microbiome may shape fetal microglia developmental trajectories near birth [55]. We found that most microglial DEGs derived from microglia could be rescued by microbial colonization. For example, changes in a panel of GABA DEGs (e.g., *Gabarap, Gabarapl2, Gabra2, Gabrb1*) were reversed by microbial recolonization, suggesting the gut microbiome may influence GABA-related signaling pathways. Our findings are consistent with several publications reporting GABA-related signaling pathways modulation through the MGB axis [15, 56, 57], although the specific cell types impacted in those studies were not delineated. Olson et al. have shown that a ketogenic diet could reduce seizures through gut-brain GABA-related pathways [15]. Cryan et al. reported that *Lactobacillus* strain ingestion regulates emotional behaviors and central GABA receptor expression in mice [56]. We have also reported that the gut microbiome may be linked with schizophrenia through the gut-brain glutamate-glutamine-GABA cycle [57]. Since microglial gene transcription was both affected by the lack of a gut microbiome, and could be rescued by microbial colonization, targeted regulation of microglial transcription through the MGB axis may represent a promising new therapeutic avenue for brain disease. In addition, we found that gut microbiome absence significantly increased microglia-astrocyte communication, which could not be reversed by microbial colonization. This result suggests that the roles of microglia-astrocyte communication in MGB-axis-related diseases need to be further explored in the future.

While we observed that microglia were most vulnerable to gut microbiome absence, single-cell technology provides an advantage to further clarify the major altered microglial subtypes. Here, we found that the gut microbiome regulated mutual transformation of microglial subpopulations, which could be effectively reversed by microbial colonization. Functionally, these altered microglial subpopulations were enriched with anti-inflammatory and Treg gene sets, suggesting changes in inflammation-related signals. In line with our findings, Shemer et al. found that peripheral inflammation induced by lipopolysaccharide (LPS) could result in microglial activation changes [58, 59]. Meanwhile, Erny et al. also observed increased LPS levels in GF mice relative to SPF mice [54]. In the future, it is necessary to further explore how the gut microbiome mediates the transformation of microglial subtypes through inflammatory pathways.

Interestingly, one of the marker genes of Hip_M1&4, *Mafb* [60], is a principal transcription factor that regulates adult microglial homeostasis. We also observed that *Runx1* [61] and *Selplg* [62], which were marker genes of PFC_M2, involved in maintaining microglial homeostasis, could be the inducer of PFC_M2. Thus, we compared our data with the research on precise typing of microglia [63] and detailed information was listed in Supplementary Table S8. Hip_M1, Hip_M4, and PFC_M2 which were enriched in GF mice showed abundant homeostatic signatures and inflammatory signatures (Supplementary Fig. 10a, b); however, Hip_M0 and PFC_M0 which were rescued by microbial colonization showed homeostatic signatures. We thought the absence of microbiome may bring disturbance to homeostatic microglia and endows it with inflammatory-related function.

In addition, using cross-species analysis, we found that transcriptome changes of microglial subpopulations regulated by gut microbiota were associated with AD and MDD. Previously, clinical and animal studies have shown that AD and MDD were accompanied by disturbances of the gut microbiome [4, 9, 13, 64]. Targeted intervention of the gut microbiota with drugs, prebiotics, and short-chain fatty acids has potential therapeutic value for these two disorders. Significantly, recent single-cell investigations have shown that AD was associated with specific changes in microglial subtypes [22–24, 53], and intervention of microglia subtypes showed a potential therapeutic effect [22]. In agreement with these findings, we found that some altered classic genes in microglia, such as *Apoe* and *Trem2*, could be reversed by microbial colonization. Corresponding to these results, we found that the absence of a gut microbiome resulted in altered short-term memory in the Y-maze test, which was also reversed by microbial colonization. A similar phenomenon was found in depression as well, while comparing the transcriptome changes of microglial subpopulations across different species including human, monkey, and mouse. These studies suggest that the gut microbiome may be involved in the pathogenesis of AD and MDD by regulating transcriptional changes of microglial subtypes.

Nonetheless, the following caveats are worth noting: (i) advent of an unfamiliar SPF in CGF group may result in a certain degree of transient stress. (ii) Like most studies [6, 10, 15, 16], we used GF mice as the main model to study brain function affected by the MGB axis. Thus, single-cell analysis of SPF mice with antibiotic treatment would further confirm our findings. (iii) In addition, spatial transcriptomic data would help further clarify the spatial localization of these vital cell-specific genes related to AD and MDD. (iv) Finally, intervening cell-specific DEGs of microglia and their subtypes also need to be explored to uncover their roles in the development of AD and MDD.

In conclusion, using an snRNA-seq approach, we showed transcriptome changes of neural cell types modulated by the absence of gut microbiome. Microglia and their subpopulations were preferentially impacted in the Hip and PFC. The gut microbiome may regulate the mutual transformation of microglial subtypes. Furthermore, their transcriptomic changes were associated with AD and MDD. Our research provided single-cell transcriptional evidence for an in-depth understanding of how the gut microbiome affects the brain, which may help uncover the pathogenesis of AD and MDD.

## Supplementary information

Supplementary Figure and Table legends

Supplementary Figures

Supplementary Table S1

Supplementary Table S2

Supplementary Table S3

Supplementary Table S4

Supplementary Table S5

Supplementary Table S6

Supplementary Table S7

Supplementary Table S8

## Data Availability

All data needed to evaluate the conclusions in the paper are present in the paper and/or the Supplementary Materials. The raw single-cell sequence data reported in this paper have been made available at the Gene Expression Omnibus (GEO) repository under the accession number GSE202704.
