# Supplementary material for: The gut microbiome modulates the transformation of microglial subtypes
Source: Mol Psychiatry. 2023 Mar 13;28(4):1611–21. doi: 10.1038/s41380-023-02017-y (PMC10208978; doi:10.1038/s41380-023-02017-y)
Supplement: Supplementary file 1 — Supplementary Figure and Table legends [file 41380_2023_2017_MOESM1_ESM.docx]

Supplementary materials

Supplementary Figure Legend

**Sup. Figure 1. Overview of the experimental approach and snRNA seq dataset.**

(a) Experimental schedule showing that the CGF group were 4-week-old (4w) GF mice cohoused with the SPF group for 4 weeks. Tissues from 8-week-old (8w) SPF, GF and CGF mice (n=3 in per group) were harvested for snRNA analysis.

(b,c) UMAP graph embedding plot depicted 72,226 nuclei divided into 36 unbiased initially identified clusters in Hip (b) and 67,698 nuclei in 29 unbiased initially identified initial clusters in PFC (c).

(d,e) Cell-type annotation was performed on the expression of well-established marker genes. Marker genes were coded according to the cell types in which they should be detected. The dot plot depicted the expression of known marker genes in 36 clusters in Hip (d) and 29 clusters in PFC (e). The size of the dots represents the percentage of expressed marker genes (Pct. exp.), and the color intensity represents the scaled average expression level (Avg. exp. scale).

**Sup. Figure 2. Gut microbiome absence influenced genes expressed in major cell types.**

(a,b) Volcano plots show DEGs (FDR< 0.05) expressed in microglia, astrocyte, oligodendrocyte, OPC, excitatory neuron and interneuron in Hip (a) and PFC (b).

(c) A total of 4999 and 6122 DEGs (FDR< 0.05) were obtained across six major cell types in Hip and PFC, respectively.

(d,e) Rose diagrams show that glial cells contributed more DEGs than neurons in Hip (d) and PFC (e).

(f,g) Mitochondrial dysfunction and RNA translation process were disturbed in major cell types by the absence of microbiome in Hip (f) and PFC (g).

**Sup. Figure 3. Cell-specific DEGs enrichment in distinguished pathways.**

(a,b) Heatmap of canonical pathways of interest enriched for cell-specific DEGs in six cell types by IPA.

**Sup. Figure 4. Gut microbiota absence mainly affects the microglial transcriptome in a brain region-specific manner.**

(a) Venn diagram depicted 370 genes shared in two regions, and 563 genes only changed in Hip, 694 in PFC.

(b) Heatmap of region-specific DEGs (GF vs SPF) enriched in top 20 canonical pathways. Opposite changes in synaptic function-related pathways showed a regional specificity in transcriptional changes caused by the lack of gut microbiome.

**Sup. Figure 5. The gut microbiome mainly modulated microglia-astrocyte communication.**

(a) Heatmaps showed cell-cell ligand/receptor-receptor pair for microglia with other cell types based on CellPhoneDB. Counts of interactions in GF and CGF demonstrated gut microbiome mainly influenced the microglia-astrocyte communication in both Hip (top) and PFC (bottom).

(b,c) Reversed genes mainly involved chemical synaptic transmission and cell adhesion in Hip (b) and protein binding and transport in PFC (c) by Gene Ontology enrichment analysis.

**Sup. Figure 6. Microglial subpopulations modulated by microbial absence were activated in the anti-inflammatory gene set.**

(a) QuSAGE analysis showed the anti-inflammatory and regulatory T cells (Treg) gene sets were most activated in Hip_M1 and Hip_M4 and inhibited in Hip_M0.

(b-d) The activity level of the anti-inflammatory and Treg gene sets in Hip_M0(b), Hip_M1(c) and Hip_M4(d).

(e) QuSAGE analysis showed that the anti-inflammatory and Treg gene sets were mostly activated in PFC_M2 and inhibited in PFC_M0. most activated gene sets and least activated gene sets of PFC_M0, PFC_M2.

(f,g) The activity level of the anti-inflammatory and Treg gene sets in PFC_M0(f) and PFC_M2(g).

**Sup. Figure 7. Anti-inflammatory and Treg genes activation in Hip_M1&4 and PFC_M2.**

(a-f) The average activity level of genes enriched in the Anti-inflammatory gene set and the Treg gene set of Hip_M1(a,b), Hip_M4(c,d) and PFC_M2(e,f).

**Sup. Figure 8. Pseudotime analysis confirmed a mutual transforming relationship between microglial subpopulations modulated by the gut microbiome.**

(a,b) In Hip(a) and PFC(b), microglial cells distribution on the pseudo-time trajectory depicted transforming direction, starting at state1 to state2 or state 3.

(c,d) Microglial cells distribution of each subcluster in Hip(e) and PFC(f) described a mutual transformation trend between Hip_M1&4 and Hip_M0, PFC_M2 and PFC_M0.

**Sup. Figure 9. Independent validation of microglial subpopulations modulated by the gut microbiome.**

(a) Cell consistency distribution of two batches of hippocampal snRNA-seq.

(b) Main cell types in Batch2 were consistent with batch1, including Microglia, Astrocyte, Oligodendrocyte, OPC, Excitatory neuron, Interneuron, and Ependymal cell, Endothelial cell, Smooth muscular cell, and Fibroblast (Endo&SMC&Fibro).

(c) Line diagram depicts high association between microglial subpopulations in batch1 and batch2. (Thickness of line means Odd ratio, Degree of red color means significance in FDR, B1_Hip_M0 versus B2_Hip_M2, OR=133.6, ***P = 3.00E-04; B1_Hip_M0 versus B2_Hip_M3, OR=91.4, ***P = 2.34E-05; B1_Hip_M1 versus B2_Hip_M0, OR=99.2, ***P =3.14E-89; B1_Hip_M4 versus B2_Hip_M0, OR=27.7, ***P = 3.90E-29; P values are from a Fisher’s exact test, FDR-BH corrected).

(d) UMAP graph showed the location of B2_Hip_M0, 2, and 3 in all microglia (batch2). Distribution among groups (SPF, GF, and CGF) shows B2_Hip_M0 decrease in SPF, moreover, increase in GF, after colonization, reverse in CGF. On the contrary, B2_Hip_M2 and 3 increase in SPF and CGF, and decrease in GF.

(e) The average proportion of the 3 subpopulations shows these microbiome-modulated subpopulations exist in mutual transformation.

**Sup. Figure 10. Exploration of subtype signature of these microglial subpopulations modulated by the gut microbiome.**

(a-b) Subtype annotation was performed on the expression of reported marker genes belonging to different signatures. The dot plot depicted the expression of genes in Hip (a) and in PFC (b). The size of the dots represents the percentage of expressed marker genes, and the color intensity represents the scaled average expression level.

Supplementary Table Legend

**Supplementary Table S1. Detailed information of single cell nucleus analysis in each sample.**

**Supplementary Table S2. Detailed information of reversed cell communication.**

**Supplementary Table S3. Detailed information of reversed gene of each cell type in Hip and PFC.**

**Supplementary Table S4. Detailed information of cross species analysis.**

**Supplementary Table S5. Detailed information of statistic information.**

**Supplementary Table S6. Detailed information of all captured genes and DEGs in each cell type.**

**Supplementary Table S7. Fisher test of microglia subpopulations in Hip between batch1 and batch2.**

**Supplement Table S8. Microglial subtype signature of captured subpopulations.**
