# Supplementary material for: The gut microbiome modulates the transformation of microglial subtypes
Source: Mol Psychiatry. 2023 Mar 13;28(4):1611–21. doi: 10.1038/s41380-023-02017-y (PMC10208978; doi:10.1038/s41380-023-02017-y)

Sup Figure 1

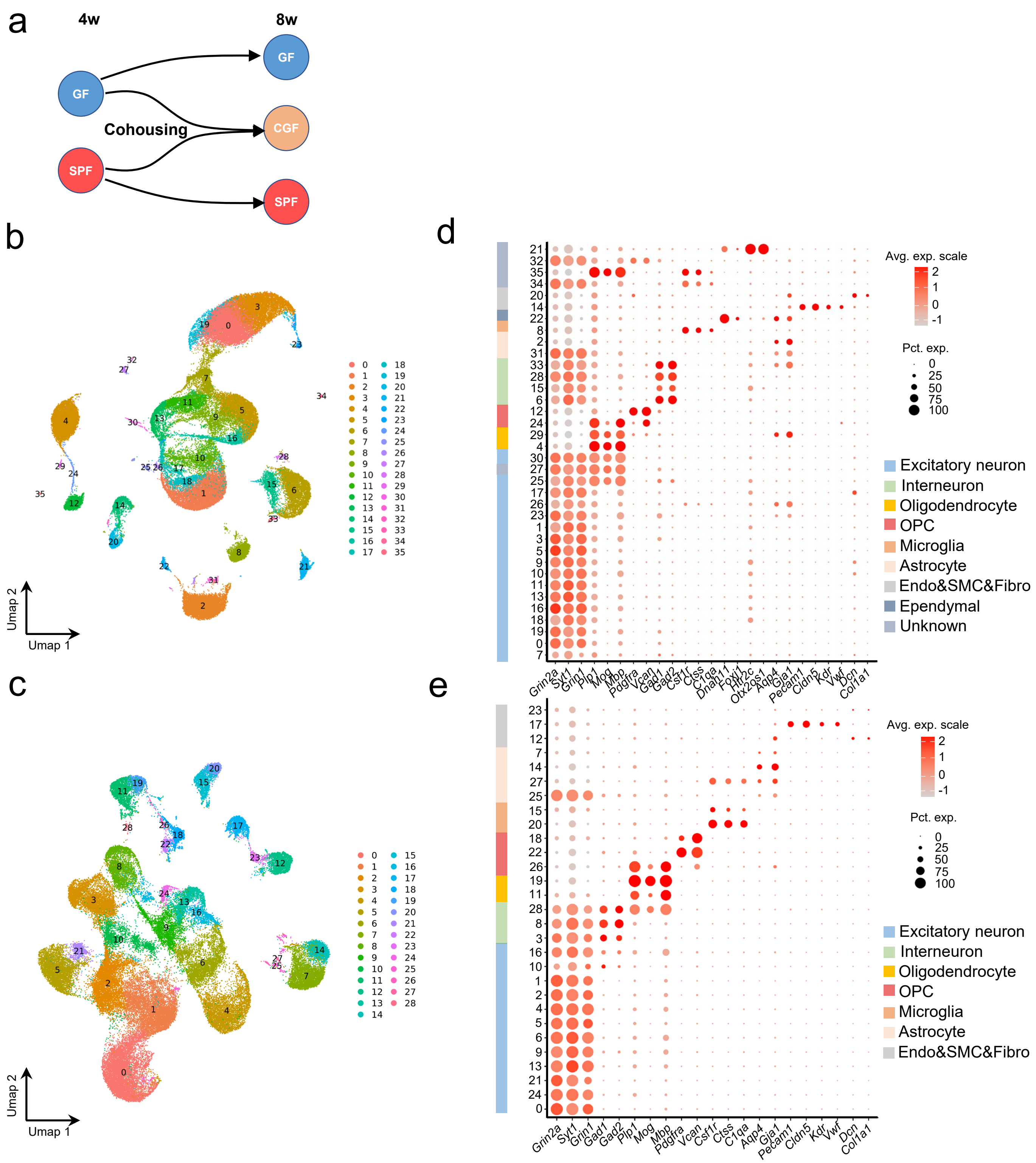

a
4w
8w
GF
GF
Cohousing
CGF
SPF
SPF
b
Umap 2
Umap 1
c
Umap 2
Umap 1
d
Avg. exp. scale
Pct. exp.
Excitatory neuron
Interneuron
Oligodendrocyte
OPC
Microglia
Astrocyte
Endo&SMC&Fibro
Ependymal
Unknown
Grin2a Syt1 Grin1 Plp1 Mog Mbp Pdgfra Vcan Gad1 Gad2 Csf1r Ctss C1qa Dnah11 Foxj1 Htr2c Otx2os1 Aqp4 Gja1 Pecam1 Cldn5 Kdr Vwf Dcn Col1a1
e
Avg. exp. scale
Pct. exp.
Excitatory neuron
Interneuron
Oligodendrocyte
OPC
Microglia
Astrocyte
Endo&SMC&Fibro
Grin2a Syt1 Grin1 Gad1 Gad2 Plp1 Mog Mbp Pdgfra Vcan Csf1r Ctss C1qa Aqp4 Gja1 Pecam1 Cldn5 Kdr Vwf Dcn Col1a1

Sup Figure 2

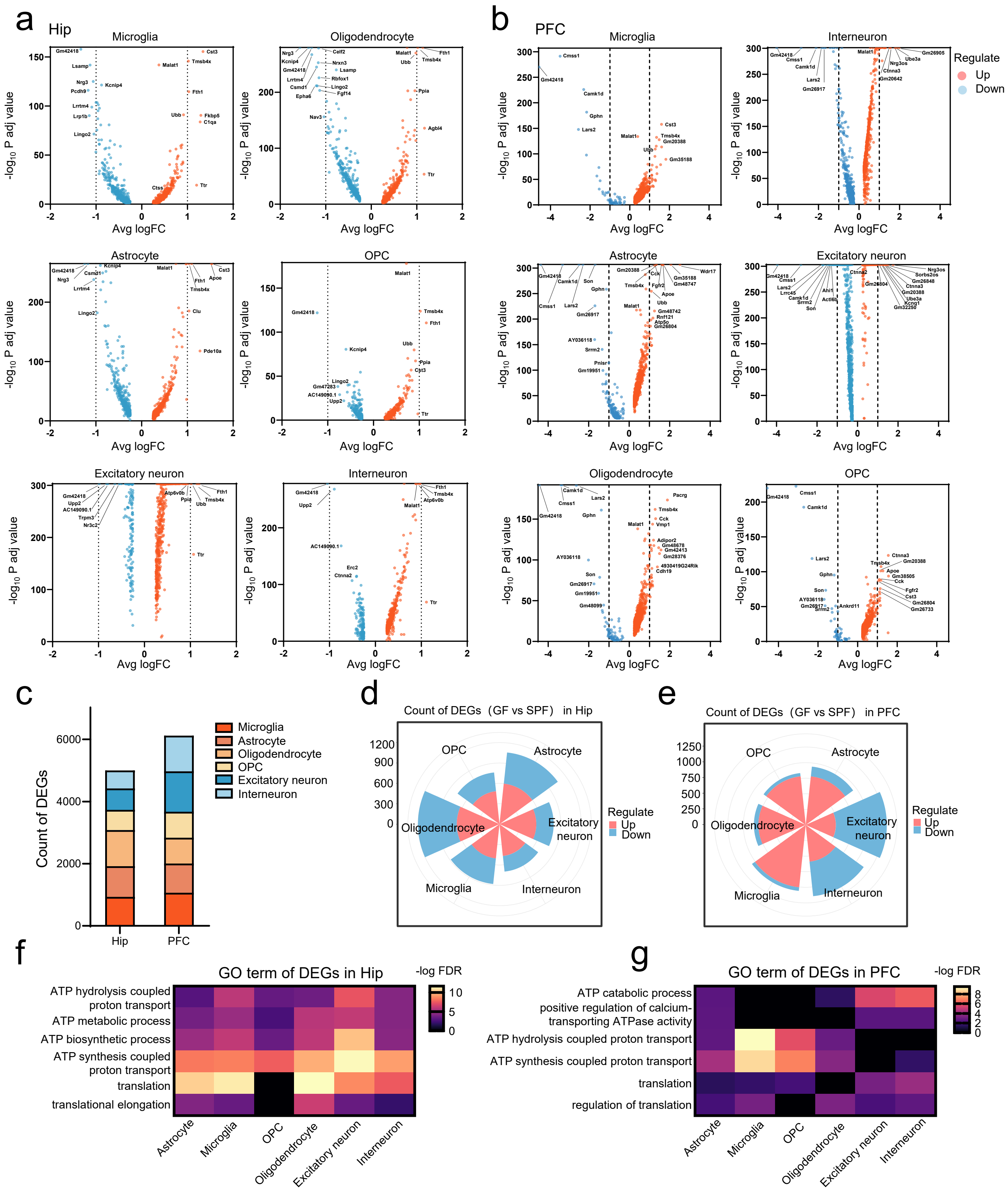
a
Hip
Microglia
Oligodendrocyte
Astrocyte
OPC
Excitatory neuron
Interneuron
b
PFC
Microglia
Interneuron
Astrocyte
Excitatory neuron
Oligodendrocyte
OPC
Regulate
Up
Down
-log10 P adj value
Avg logFC
c
Count of DEGs
Hip
PFC
Microglia
Astrocyte
Oligodendrocyte
OPC
Excitatory neuron
Interneuron
d
Count of DEGs (GF vs SPF) in Hip
e
Count of DEGs (GF vs SPF) in PFC
f
GO term of DEGs in Hip
-log FDR
ATP hydrolysis coupled proton transport
ATP metabolic process
ATP biosynthetic process
ATP synthesis coupled proton transport
translation
translational elongation
g
GO term of DEGs in PFC
ATP catabolic process
positive regulation of calcium-transporting ATPase activity
ATP hydrolysis coupled proton transport
ATP synthesis coupled proton transport
translation
regulation of translation
Astrocyte
Microglia
OPC
Oligodendrocyte
Excitatory neuron
Interneuron

Sup Figure 3

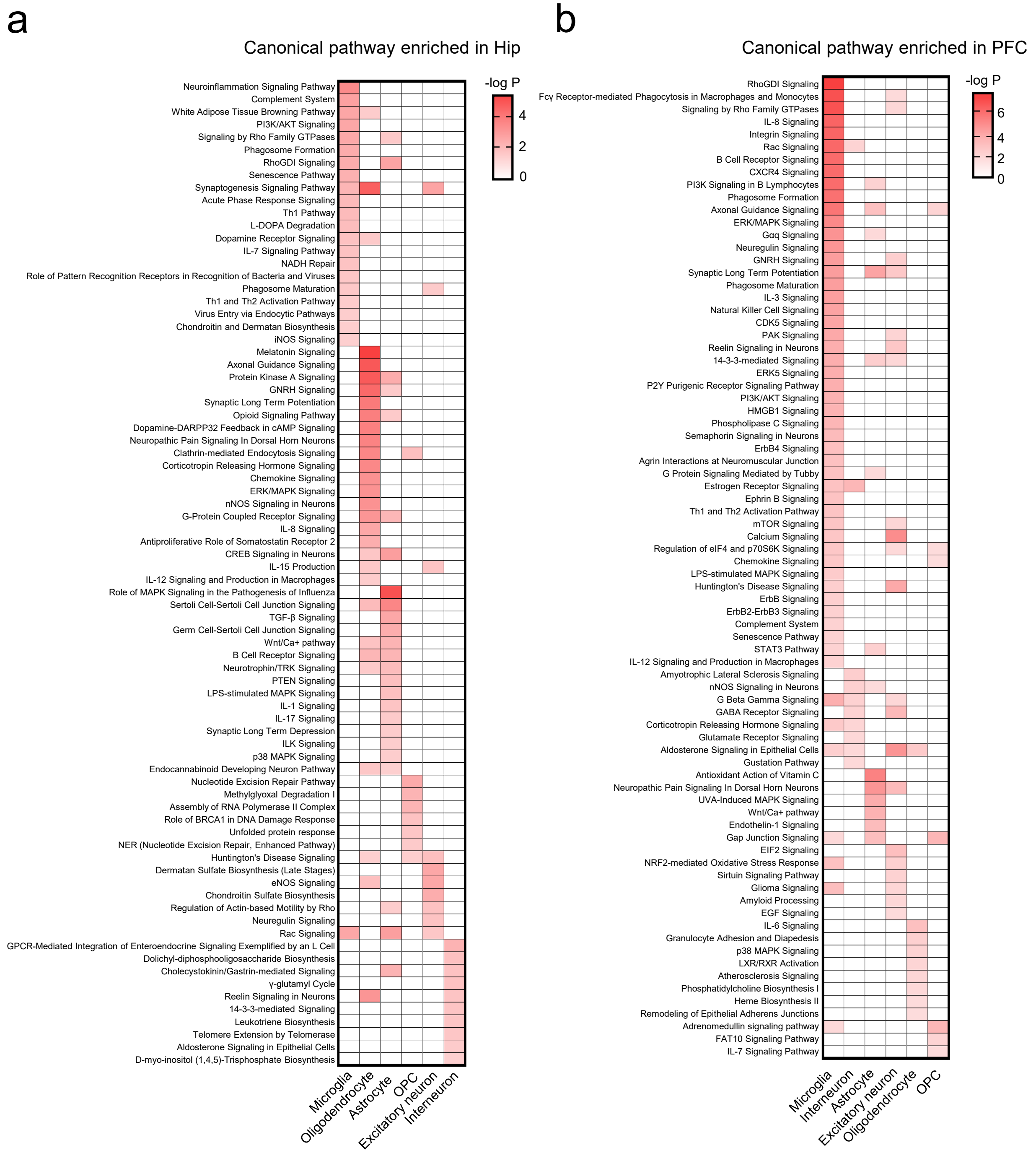
a
Canonical pathway enriched in Hip
-log P
4
2
0
Neuroinflammation Signaling Pathway
Complement System
White Adipose Tissue Browning Pathway
PI3K/AKT Signaling
Signaling by Rho Family GTPases
Phagosome Formation
RhoGDI Signaling
Senescence Pathway
Synaptogenesis Signaling Pathway
Acute Phase Response Signaling
Th1 Pathway
L-DOPA Degradation
Dopamine Receptor Signaling
IL-7 Signaling Pathway
NADH Repair
Role of Pattern Recognition Receptors in Recognition of Bacteria and Viruses
Phagosome Maturation
Th1 and Th2 Activation Pathway
Virus Entry via Endocytic Pathways
Chondroitin and Dermatan Biosynthesis
iNOS Signaling
Melatonin Signaling
Axonal Guidance Signaling
Protein Kinase A Signaling
GNRH Signaling
Synaptic Long Term Potentiation
Opioid Signaling Pathway
Dopamine-DARPP32 Feedback in cAMP Signaling
Neuropathic Pain Signaling In Dorsal Horn Neurons
Clathrin-mediated Endocytosis Signaling
Corticotropin Releasing Hormone Signaling
Chemokine Signaling
ERK/MAPK Signaling
nNOS Signaling in Neurons
G-Protein Coupled Receptor Signaling
IL-8 Signaling
Antiproliferative Role of Somatostatin Receptor 2
CREB Signaling in Neurons
IL-15 Production
IL-12 Signaling and Production in Macrophages
Role of MAPK Signaling in the Pathogenesis of Influenza
Sertoli Cell-Sertoli Cell Junction Signaling
TGF-β Signaling
Germ Cell-Sertoli Cell Junction Signaling
Wnt/Ca+ pathway
B Cell Receptor Signaling
Neurotrophin/TRK Signaling
PTEN Signaling
LPS-stimulated MAPK Signaling
IL-1 Signaling
IL-17 Signaling
Synaptic Long Term Depression
ILK Signaling
p38 MAPK Signaling
Endocannabinoid Developing Neuron Pathway
Nucleotide Excision Repair Pathway
Methylglyoxal Degradation I
Assembly of RNA Polymerase II Complex
Role of BRCA1 in DNA Damage Response
Unfolded protein response
NER (Nucleotide Excision Repair, Enhanced Pathway)
Huntington's Disease Signaling
Dermatan Sulfate Biosynthesis (Late Stages)
eNOS Signaling
Chondroitin Sulfate Biosynthesis
Regulation of Actin-based Motility by Rho
Neuregulin Signaling
Rac Signaling
GPCR-Mediated Integration of Enteroendocrine Signaling Exemplified by an L Cell
Dolichyl-diphosphooligosaccharide Biosynthesis
Cholecystokinin/Gastrin-mediated Signaling
γ-glutamyl Cycle
Reelin Signaling in Neurons
14-3-3-mediated Signaling
Leukotriene Biosynthesis
Telomere Extension by Telomerase
Aldosterone Signaling in Epithelial Cells
D-myo-inositol (1,4,5)-Trisphosphate Biosynthesis
Microglia
Oligodendrocyte
Astrocyte
OPC
Excitatory neuron
Interneuron
b
Canonical pathway enriched in PFC
-log P
6
4
2
0
RhoGDI Signaling
Fcγ Receptor-mediated Phagocytosis in Macrophages and Monocytes
Signaling by Rho Family GTPases
IL-8 Signaling
Integrin Signaling
Rac Signaling
B Cell Receptor Signaling
CXCR4 Signaling
PI3K Signaling in B Lymphocytes
Phagosome Formation
Axonal Guidance Signaling
ERK/MAPK Signaling
Gαq Signaling
Neuregulin Signaling
GNRH Signaling
Synaptic Long Term Potentiation
Phagosome Maturation
IL-3 Signaling
Natural Killer Cell Signaling
CDK5 Signaling
PAK Signaling
Reelin Signaling in Neurons
14-3-3-mediated Signaling
ERK5 Signaling
P2Y Purigenic Receptor Signaling Pathway
PI3K/AKT Signaling
HMGB1 Signaling
Phospholipase C Signaling
Semaphorin Signaling in Neurons
ErbB4 Signaling
Agrin Interactions at Neuromuscular Junction
G Protein Signaling Mediated by Tubby
Estrogen Receptor Signaling
Ephrin B Signaling
Th1 and Th2 Activation Pathway
mTOR Signaling
Calcium Signaling
Regulation of eIF4 and p70S6K Signaling
Chemokine Signaling
LPS-stimulated MAPK Signaling
Huntington's Disease Signaling
ErbB Signaling
ErbB2-ErbB3 Signaling
Complement System
Senescence Pathway
STAT3 Pathway
IL-12 Signaling and Production in Macrophages
Amyotrophic Lateral Sclerosis Signaling
nNOS Signaling in Neurons
G Beta Gamma Signaling
GABA Receptor Signaling
Corticotropin Releasing Hormone Signaling
Glutamate Receptor Signaling
Aldosterone Signaling in Epithelial Cells
Gustation Pathway
Antioxidant Action of Vitamin C
Neuropathic Pain Signaling In Dorsal Horn Neurons
UVA-Induced MAPK Signaling
Wnt/Ca+ pathway
Endothelin-1 Signaling
Gap Junction Signaling
EIF2 Signaling
NRF2-mediated Oxidative Stress Response
Sirtuin Signaling Pathway
Glioma Signaling
Amyloid Processing
EGF Signaling
IL-6 Signaling
Granulocyte Adhesion and Diapedesis
p38 MAPK Signaling
LXR/RXR Activation
Atherosclerosis Signaling
Phosphatidylcholine Biosynthesis I
Heme Biosynthesis II
Remodeling of Epithelial Adherens Junctions
Adrenomedullin signaling pathway
FAT10 Signaling Pathway
IL-7 Signaling Pathway
Microglia
Interneuron
Astrocyte
Excitatory neuron
Oligodendrocyte
OPC

Sup Figure 4

a

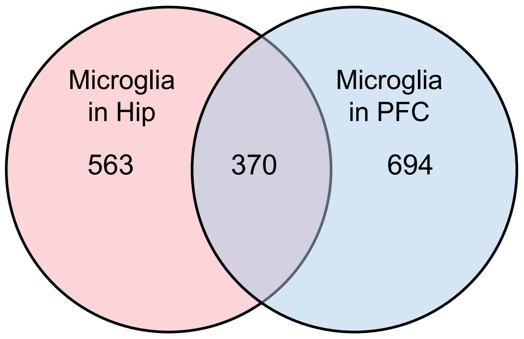

b

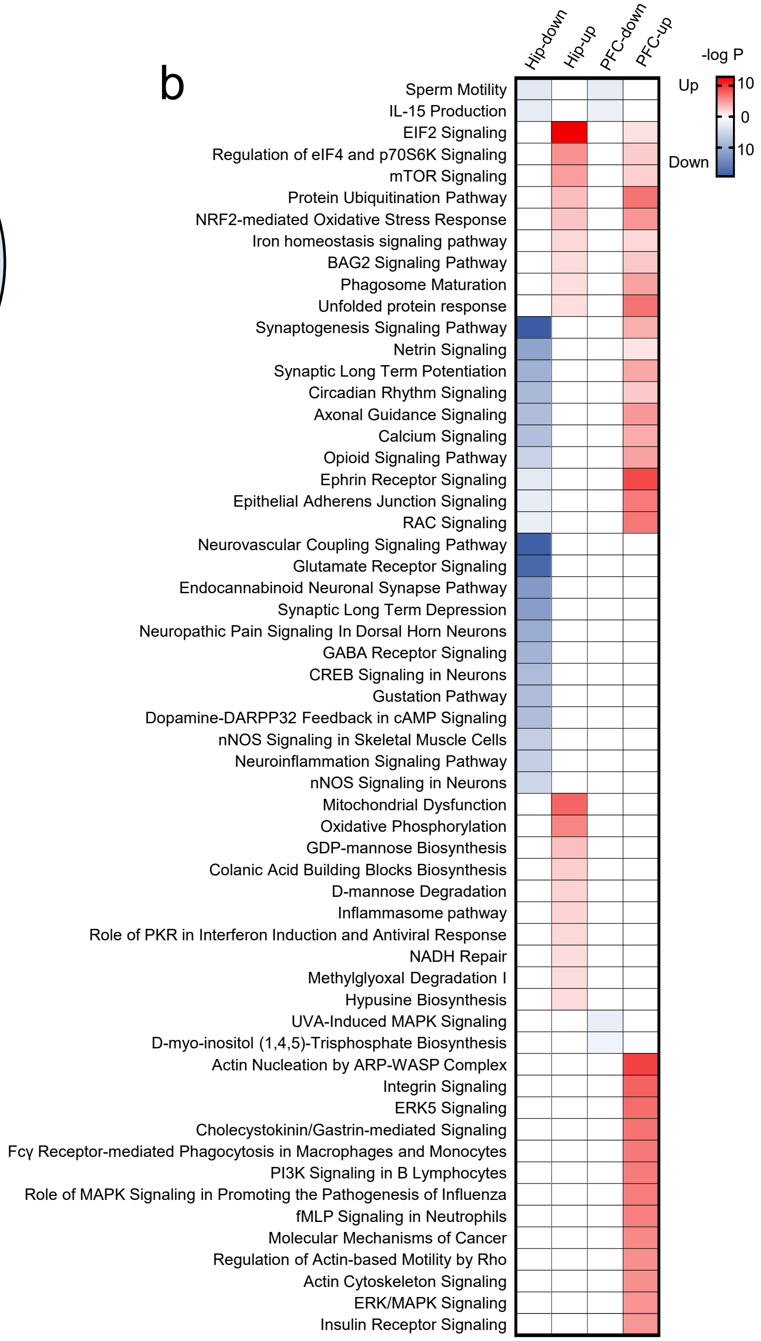

Sup Figure 5

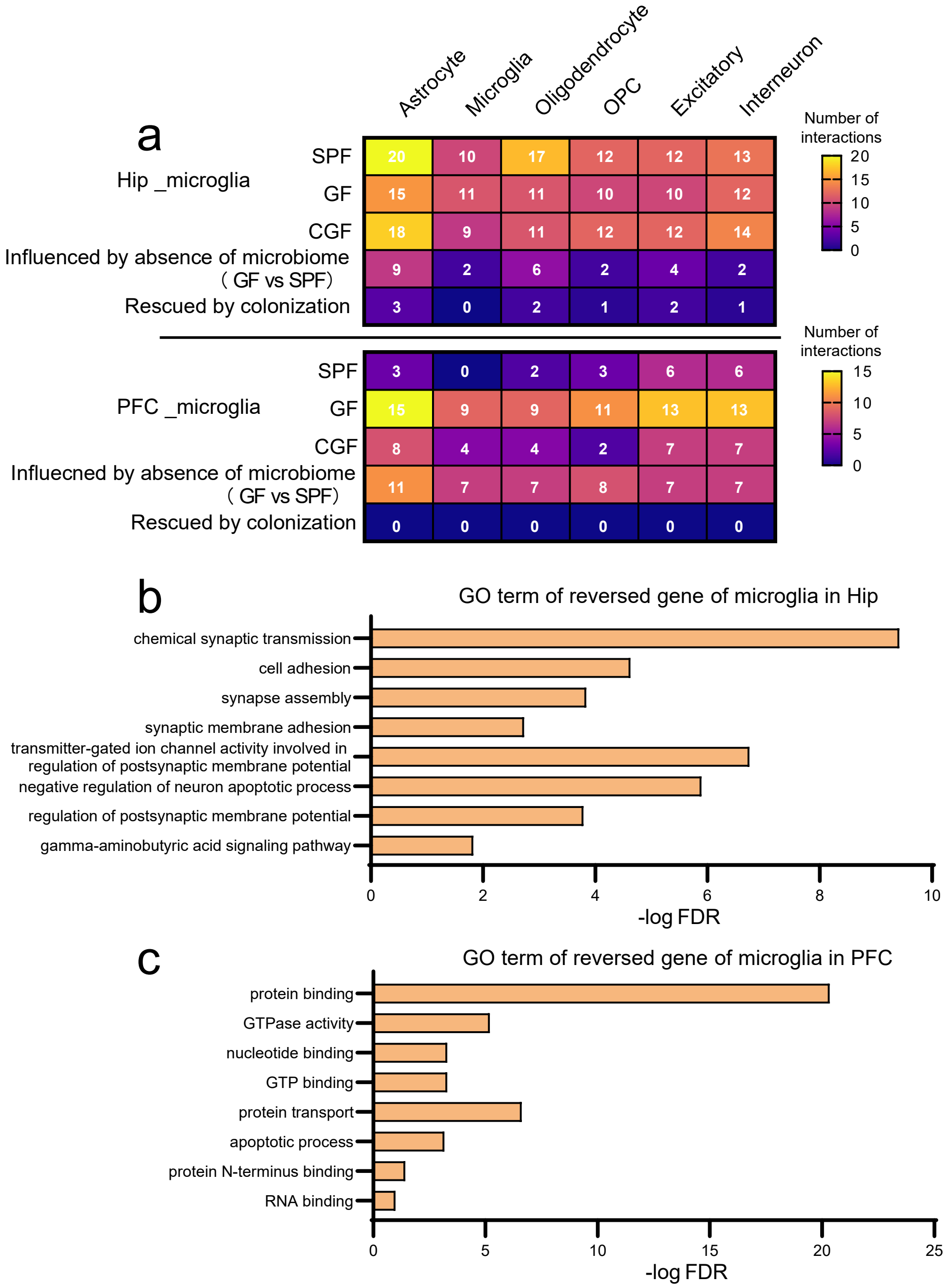
a
Astrocyte
Microglia
Oligodendrocyte
OPC
Excitatory
Interneuron
Hip _microglia
SPF
GF
CGF
Influenced by absence of microbiome
(GF vs SPF)
Rescued by colonization
20 10 17 12 12 13
15 11 11 10 10 12
18 9 11 12 12 14
9 2 6 2 4 2
3 0 2 1 2 1
Number of interactions
20
15
10
5
0
PFC _microglia
SPF
GF
CGF
Influecned by absence of microbiome
(GF vs SPF)
Rescued by colonization
3 0 2 3 6 6
15 9 9 11 13 13
8 4 4 2 7 7
11 7 7 8 7 7
0 0 0 0 0 0
Number of interactions
15
10
5
0
b
GO term of reversed gene of microglia in Hip
chemical synaptic transmission
cell adhesion
synapse assembly
synaptic membrane adhesion
transmitter-gated ion channel activity involved in regulation of postsynaptic membrane potential
negative regulation of neuron apoptotic process
regulation of postsynaptic membrane potential
gamma-aminobutyric acid signaling pathway
0
2
4
6
8
10
-log FDR
c
GO term of reversed gene of microglia in PFC
protein binding
GTPase activity
nucleotide binding
GTP binding
protein transport
apoptotic process
protein N-terminus binding
RNA binding
0
5
10
15
20
25
-log FDR

Sup Figure 6

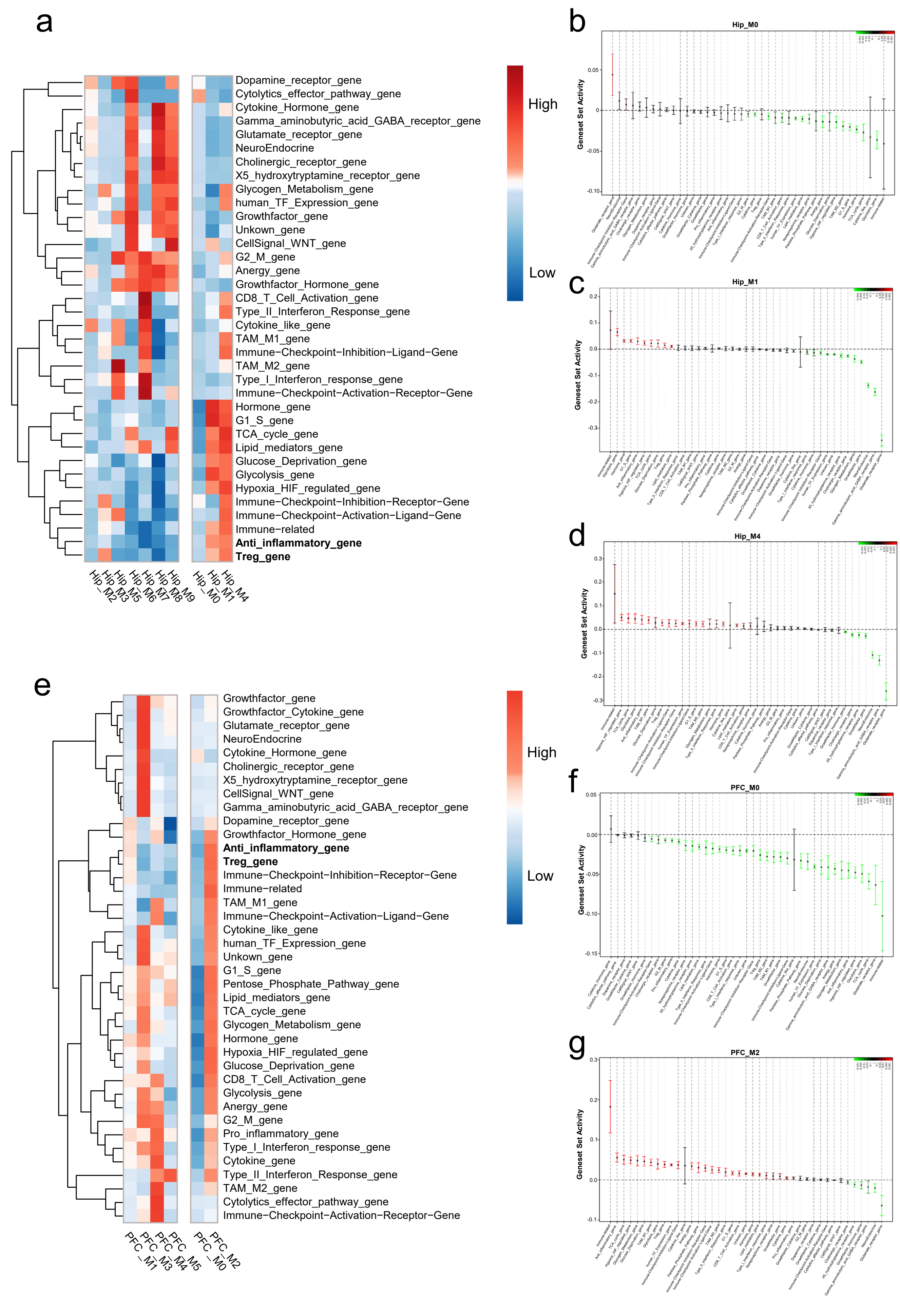
a
Dopamine_receptor_gene
Cytolytics_effector_pathway_gene
Cytokine_Hormone_gene
Gamma_aminobutyric_acid_GABA_receptor_gene
Glutamate_receptor_gene
NeuroEndocrine
Cholinergic_receptor_gene
X5_hydroxytryptamine_receptor_gene
Glycogen_Metabolism_gene
human_TF_Expression_gene
Growthfactor_gene
Unkown_gene
CellSignal_WNT_gene
G2_M_gene
Anergy_gene
Growthfactor_Hormone_gene
CD8_T_Cell_Activation_gene
Type_II_Interferon_Response_gene
Cytokine_like_gene
TAM_M1_gene
Immune−Checkpoint−Inhibition−Ligand−Gene
TAM_M2_gene
Type_I_Interferon_response_gene
Immune−Checkpoint−Activation−Receptor−Gene
Hormone_gene
G1_S_gene
TCA_cycle_gene
Lipid_mediators_gene
Glucose_Deprivation_gene
Glycolysis_gene
Hypoxia_HIF_regulated_gene
Immune−Checkpoint−Inhibition−Receptor−Gene
Immune−Checkpoint−Activation−Ligand−Gene
Immune−related
Anti_inflammatory_gene
Treg_gene
Hip_M2
Hip_M3
Hip_M5
Hip_M6
Hip_M7
Hip_M8
Hip_M9
Hip_M0
Hip_M1
Hip_M4
High
Low
b
Hip_M0
Geneset Set Activity
c
Hip_M1
Geneset Set Activity
d
Hip_M4
Geneset Set Activity
e
Growthfactor_gene
Growthfactor_Cytokine_gene
Glutamate_receptor_gene
NeuroEndocrine
Cytokine_Hormone_gene
Cholinergic_receptor_gene
X5_hydroxytryptamine_receptor_gene
CellSignal_WNT_gene
Gamma_aminobutyric_acid_GABA_receptor_gene
Dopamine_receptor_gene
Growthfactor_Hormone_gene
Anti_inflammatory_gene
Treg_gene
Immune−Checkpoint−Inhibition−Receptor−Gene
Immune−related
TAM_M1_gene
Immune−Checkpoint−Activation−Ligand−Gene
Cytokine_like_gene
human_TF_Expression_gene
Unkown_gene
G1_S_gene
Pentose_Phosphate_Pathway_gene
Lipid_mediators_gene
TCA_cycle_gene
Glycogen_Metabolism_gene
Hormone_gene
Hypoxia_HIF_regulated_gene
Glucose_Deprivation_gene
CD8_T_Cell_Activation_gene
Glycolysis_gene
Anergy_gene
G2_M_gene
Pro_inflammatory_gene
Type_I_Interferon_response_gene
Cytokine_gene
Type_II_Interferon_Response_gene
TAM_M2_gene
Cytolytics_effector_pathway_gene
Immune−Checkpoint−Activation−Receptor−Gene
PFC_M1
PFC_M3
PFC_M4
PFC_M5
PFC_M0
PFC_M2
High
Low
f
PFC_M0
Geneset Set Activity
g
PFC_M2
Geneset Set Activity

Sup Figure 7

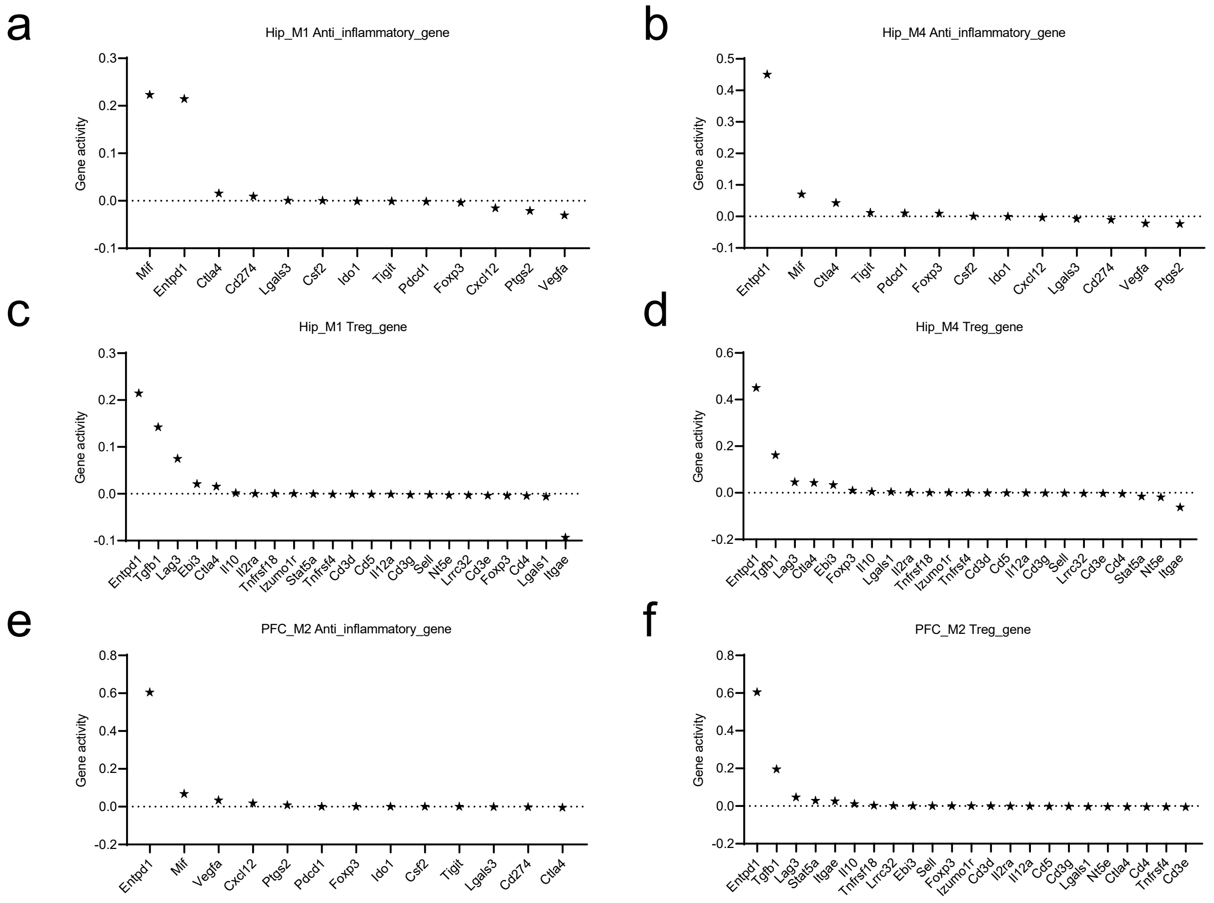
a
Hip_M1 Anti_inflammatory_gene
Gene activity
0.3
0.2
0.1
0.0
-0.1
Mif Entpd1 Ctla4 Cd274 Lgals3 Csf2 Ido1 Tigit Pdcd1 Foxp3 Cxcl12 Ptgs2 Vegfa
b
Hip_M4 Anti_inflammatory_gene
Gene activity
0.5
0.4
0.3
0.2
0.1
0.0
-0.1
Entpd1 Mif Ctla4 Tigit Pdcd1 Foxp3 Csf2 Ido1 Cxcl12 Lgals3 Cd274 Vegfa Ptgs2
c
Hip_M1 Treg_gene
Gene activity
0.3
0.2
0.1
0.0
-0.1
Entpd1 Tgfb1 Lag3 Ebi3 Ctla4 Il10 Il2ra Tnfrsf18 Izumo1r Stat5a Tnfrsf4 Cd3d Cd5 Il12a Cd3g Sell Nt5e Lrrc32 Cd3e Foxp3 Cd4 Lgals1 Itgae
d
Hip_M4 Treg_gene
Gene activity
0.6
0.4
0.2
0.0
-0.2
Entpd1 Tgfb1 Lag3 Ctla4 Ebi3 Foxp3 Il10 Lgals1 Il2ra Tnfrsf18 Izumo1r Tnfrsf4 Cd3d Cd5 Il12a Cd3g Sell Lrrc32 Cd3e Cd4 Stat5a Nt5e Itgae
e
PFC_M2 Anti_inflammatory_gene
Gene activity
0.8
0.6
0.4
0.2
0.0
-0.2
Entpd1 Mif Vegfa Cxcl12 Ptgs2 Pdcd1 Foxp3 Ido1 Csf2 Tigit Lgals3 Cd274 Ctla4
f
PFC_M2 Treg_gene
Gene activity
0.8
0.6
0.4
0.2
0.0
-0.2
Entpd1 Tgfb1 Lag3 Stat5a Itgae Il10 Tnfrsf18 Lrrc32 Ebi3 Sell Foxp3 Izumo1r Cd3d Il2ra Il12a Cd5 Cd3g Lgals1 Nt5e Ctla4 Cd4 Tnfrsf4 Cd3e

Sup Figure 8

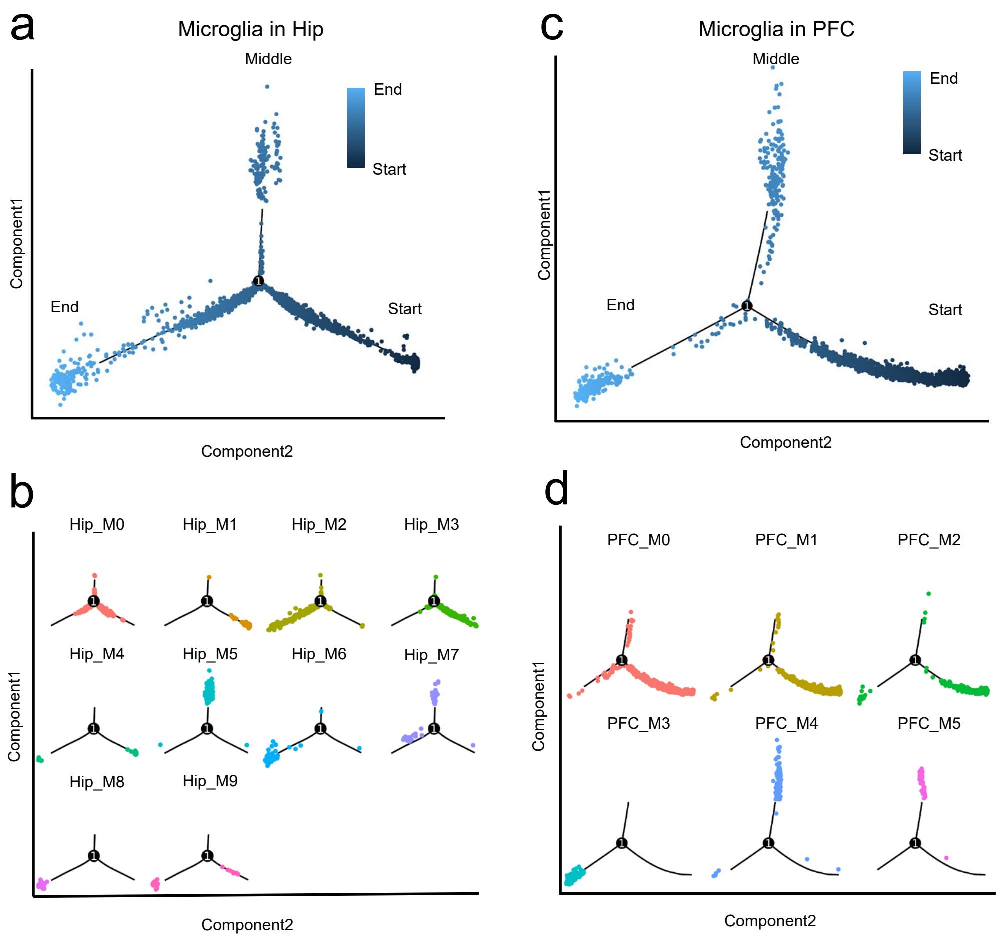
a
Microglia in Hip
Middle
End
Start
Component1
End
Start
Component2
c
Microglia in PFC
Middle
End
Start
Component1
End
Start
Component2
b
Hip_M0
Hip_M1
Hip_M2
Hip_M3
Hip_M4
Hip_M5
Hip_M6
Hip_M7
Hip_M8
Hip_M9
Component1
Component2
d
PFC_M0
PFC_M1
PFC_M2
PFC_M3
PFC_M4
PFC_M5
Component1
Component2

Sup Figure 9

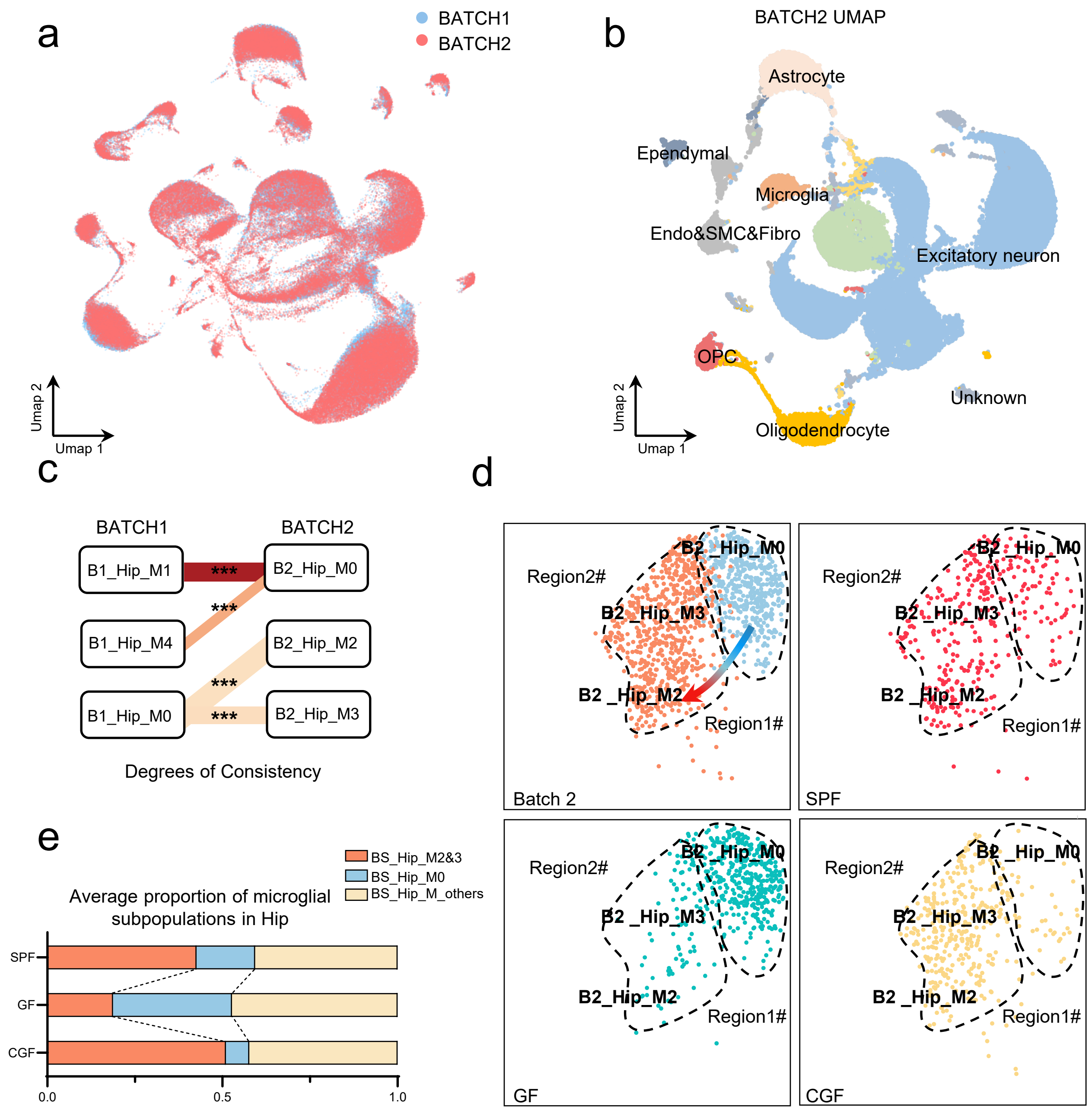

a
BATCH1
BATCH2
Umap 2
Umap 1
b
BATCH2 UMAP
Astrocyte
Ependymal
Microglia
Endo&SMC&Fibro
Excitatory neuron
OPC
Unknown
Oligodendrocyte
Umap 2
Umap 1
c
BATCH1
BATCH2
B1_Hip_M1
B2_Hip_M0
B1_Hip_M4
B2_Hip_M2
B1_Hip_M0
B2_Hip_M3
***
***
***
***
Degrees of Consistency
d
Region2#
B2 _Hip_M0
B2 _Hip_M3
B2 _Hip_M2
Region1#
Batch 2
SPF
GF
CGF
e
BS_Hip_M2&3
BS_Hip_M0
BS_Hip_M_others
Average proportion of microglial subpopulations in Hip
SPF
GF
CGF
0.0
0.5
1.0

Sup Figure 10

a

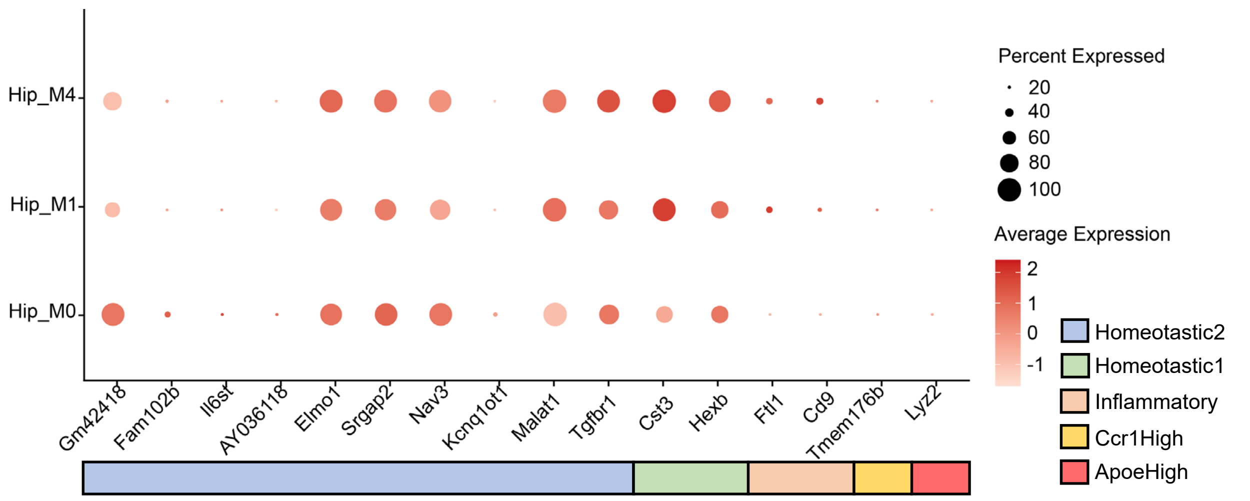

b

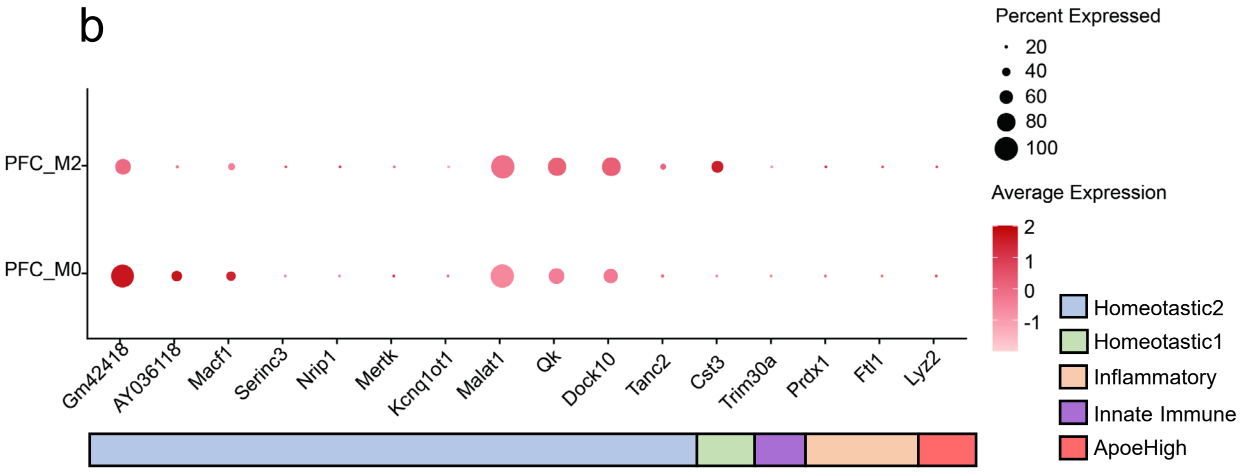

Supplement: Supplementary file 2 — Supplementary Figures [file 41380_2023_2017_MOESM2_ESM.pdf]
